# Safety and Efficacy of Bleb Needling with Antimetabolite after Trabeculectomy Failure in Glaucoma Patients: A Systemic Review and Meta-Analysis

**DOI:** 10.1155/2020/4310258

**Published:** 2020-11-30

**Authors:** Xuhao Chen, Lingge Suo, Ying Hong, Chun Zhang

**Affiliations:** ^1^Department of Ophthalmology, Peking University Third Hospital, Beijing, China; ^2^Beijing Key Laboratory of Restoration of Damaged Ocular Nerve, Peking University Third Hospital, Beijing, China

## Abstract

**Background:**

Bleb needling with subconjunctival injection of antimetabolites had become a widely accepted approach for trabeculectomy failure. However, IOP reduction effects, success rates, and complications occurrence for this procedure showed great inconsistency among the different studies.

**Methods:**

We conducted a literature search on PubMed, Embase, Cochrane Library, and ClinicalTrials.gov. A random-effects model was performed on the extracted data based on the included studies. The intraocular pressure (IOP) and number of antiglaucomatous medications before and after the surgery were pooled for meta-analysis. The success and complication rates were estimated based on the results. Subgroup analysis, sensitivity analysis, and metaregression were applied to explore the origination of heterogeneity.

**Results:**

Thirty-seven studies with a total of 2182 patients were finally included in our review. For the present meta-analysis, the overall effects of bleb needling at the last visit revealed a reduction in IOP of 9.74 mmHg (95% confidence interval (CI) [8.85, 10.63]), 45.9% (95% CI [39.0%, 53.0%]) for complete success rate, and 70.4% (95% CI [63.5%, 77.0%]) for qualified success rate. Application of mitomycin C (MMC) and 5-fluorouracil (5-Fu) during the procedure were efficacious for IOP control during the follow-up. Metaregression revealed that possible origination of heterogeneity was baseline IOP before bleb needling, revealing a trend that higher baseline IOP correlated with a greater IOP reduction results (*p* < 0.001). For safety profile, conjunctival haemorrhage (5.7%, 95% CI [2.5%, 10.1%]), hyphema (5.5%, 95% CI [3.0%, 8.7%]), and bleb leakage (5.0%, 95% CI [3.2%, 7.3%]) had the highest estimate of incidence. An increasing number of needling was the main risk factor for needling failure.

**Conclusion:**

Bleb needling with antimetabolites could be considered an effective and safe procedure after trabeculectomy failure. After the process, patients will gain IOP control and reduce antiglaucomatous medications for at least six months with 5-Fu or MMC. Meanwhile, the overall estimates for complications were relatively low in the whole process.

## 1. Introduction

Trabeculectomy is a traditional filtering surgery designed to control intraocular pressure (IOP) with long-term efficacy [1]. With fibrosis and scar formation under the conjunctival and episcleral interface of the filtering bleb, however, IOP is likely to lose control at different follow-up points after the surgery [2]. Bleb needling was designed to rebuild the failing blebs and resolve the unfunctional drainage channel with relatively small injuries. In a setting of operating room or slit-lamp at the clinics, a needle was incised into the subconjunctival space to dissect the adhesion by sweeping motions. Further incision into the anterior chamber may be applied to reopen the scleral flap. Typically, bleb needling with subconjunctival injection of antimetabolites had become a widely accepted approach for the failure of trabeculectomy since the introduction of 5-fluorouracil (5-Fu) [3] and mitomycin C (MMC) [4].

Following the previously mentioned principles, different glaucoma specialists may change the details of practices, according to their own experience [5]. Vast differences were shown in the methodology and clinical settings for the studies of bleb needling in the literature. To our knowledge, there were only limited studies about overall estimates for bleb needing with antimetabolites. A previous systemic review about bleb needling without antimetabolites found no conclusive evidence that a single needling outstood conservative antiglaucomatous medication in IOP controlling for encapsulated blebs [6]. As antimetabolites had been used widely, the strict limitation to bleb morphology and lack of updated data had weakened the clinical value of the systemic review. Furthermore, IOP reduction level and success rates after bleb needling varied considerably among studies [2, 4, 7–41]. Comparison and analysis of the possible factors and influence of these between-study variances can strengthen our understanding of the appropriate clinical setting for bleb needling. Therefore, our meta-analysis aimed to shed light on the bleb needling with antimetabolite application after trabeculectomy failure to evaluate the overall IOP reduction effects and complications. To be specific, subscales of antiglaucomatous medications usages, success rates under different definitions, risk factors for failure, and bleb characteristics were simultaneously assessed to provide a more comprehensive view of the safety and efficacy profile of bleb needling.

## 2. Methods

### 2.1. Search Strategy

The meta-analysis was conducted following the PRISMA guidelines [42]. We performed a systematic electronic search of PubMed, Embase, Cochrane Library, and ClinicalTrials.gov. for articles published until February 2020. The keywords for our search included glaucoma, filtering surgery, trabeculectomy, bleb needling, needling revision, failed bleb, and encapsulated bleb. Relevant publications were examined for references until no further studies were found.

### 2.2. Selection Criteria

#### 2.2.1. Inclusion Criteria

1. Patients diagnosed as having glaucoma, regardless of age, ethnicity, race, or sex; those who underwent bleb needling surgery after trabeculectomy or phacotrabeculectomy failure.
2. The intervention of bleb needling with subconjunctival injection of antimetabolites surgery due to uncontrolled IOP after trabeculectomy.
3. The study evaluating our primary outcomes of intraocular pressure before and after bleb needling, or success rate at one year or last visit follow-up.
4. Studies should be either a randomized-control trial or an observational study. Both comparative and noncomparative studies were included.

#### 2.2.2. Exclusion Criteria

1. Reviews, case reports, non-English studies, published abstracts without available full texts, or reports from meetings
2. The previous intervention of any drainage implantation, nonpenetrating filter surgery
3. Bleb needling was not the primary intervention; extra approaches other than bleb needling and subconjunctival injection of medication, or simply an injection of medication without bleb needling will be excluded
4. Studies about the usage of medications other than a subconjunctival injection of antimetabolites; unclear data of usage were excluded
5. Studies population from the same trial
6. Follow-up <6 months or unknown
7. <10 people in a group

### 2.3. Outcome Measures

#### 2.3.1. Primary Outcomes

The primary outcomes were mean intraocular pressure (IOP) measured in mmHg and success rate at the last visit of the follow-up.

#### 2.3.2. Secondary Outcomes

The secondary outcomes were as follows:

1. Mean IOP and success rate at different time points
2. The number of antiglaucoma medications
3. The proportion of participants presenting with a specific adverse event because of bleb needling
4. Bleb morphology changes
5. Risk factors for bleb needling failure

### 2.4. Data Extraction

Two reviewers (XC and YH) independently read each title and abstract to exclude irrelevant studies and then reviewed each full text to ascertain its eligibility and to extract data. Any disagreements were resolved by other authors (LS and CZ) and a consensus was reached among all the reviewers. Authors were contacted, if necessary, to acquire any missing information and for unpublished studies.

We extracted data representing the efficacy and safety of bleb needling, including primary outcomes and secondary outcomes. Study characteristics and patients' baseline characteristics were extracted, as well. Different arms for comparative study for MMC and 5-Fu were distributed to different subgroups.

### 2.5. Risk of Bias Assessment

The risk of bias of studies was evaluated using the checklist recommended by the Agency for Healthcare Research and Quality (AHRQ) [43]. Based on the reviewers' judgment, for each domain (including selection bias, performance bias, detection bias, attrition bias, and reporting bias) on the checklist, every article was rated as having a “low,” “high,” or “unclear” risk of bias in each domain. Data organization was done using RevMan version 5.3 (Cochrane Community, London, UK).

### 2.6. Data Synthesis and Analytical Methods

Data were summarized, and the meta-analysis was performed using Stata version 14.0 (StataCorp, College Station, TX). The IOP and the number of glaucoma medications before and after the bleb needling were compared with a weighted mean difference (WMD). Estimates of complication and success rates were aggregated with double arcsine transmission during our meta-analysis. All the indices were analyzed in a random-effects model, and data were presented as mean with 95% confidence interval (CI). Besides, we described the heterogeneity by calculating *I*^2^ values among the studies, which ranged from 0% to 100% (with 25%, 50%, and 75% representing low, moderate, and high heterogeneity, resp.). Subgroup analyses were conducted according to antimetabolites, study design, countries or regions, bleb morphology, surgery condition, and acceptance for repeating needling. Metaregression with restricted maximum likelihood (REML) method and Hartung–Knapp method were applied to explore the possible source of heterogeneity. When median with range and size were available in the original articles, data were transformed into mean with standard deviation values as described by Hozo et al. [44]. To detect publication biases, we created funnel plots where asymmetry indicated publication bias. Egger's statistical tests of funnel plot asymmetry were also applied. Sensitivity analysis was performed to evaluate the effect of a single trial on the overall pooled estimate by omitting one trial in each turn. *p* < 0.05 was considered statistically significant. Results of our meta-analysis were partially converted to graphs created using Prism 7.0 (GraphPad Software, La Jolla, CA).

## 3. Results

### 3.1. Search Results

**Figure 1:**
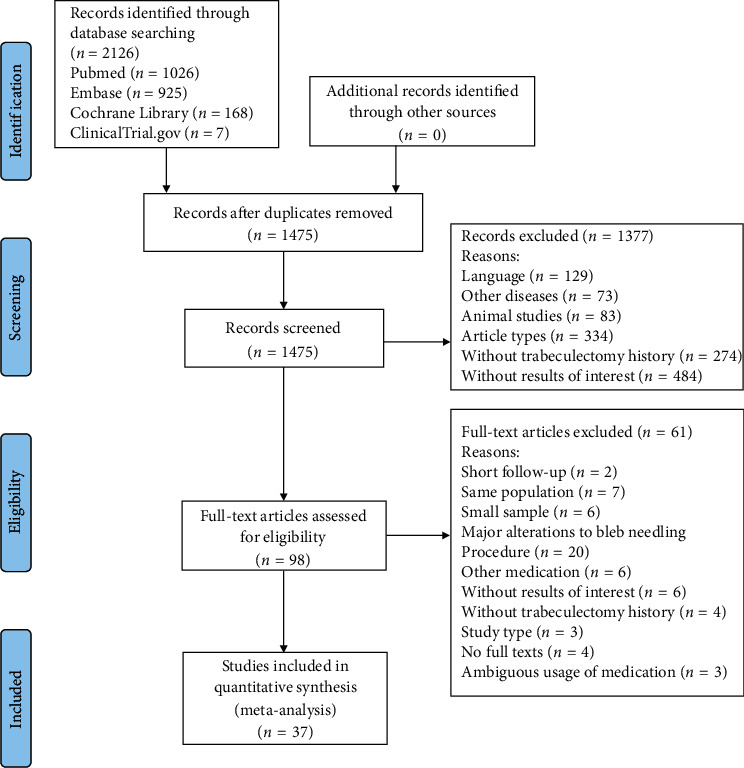
Flow diagram of literature search and included studies for meta-analysis.

A total of 2126 studies were identified based on the search strategy. After duplication removal, we reviewed the titles and abstracts of 1475 articles and excluded 1377 studies with reasons. The full-text screening was performed for the remaining studies, and finally, 37 full-text articles met the eligibility criteria for meta-analysis.  Figure 1 demonstrates the flow diagram of the literature search process.

### 3.2. Characteristics of Included Studies

The total number of eyes included was 2182 dating from 1993 to 2019. Specifically, 1333 eyes had undergone bleb needling with 5-Fu, and 849 eyes, with MMC. Of 37 studies included, there were 25 noncomparative case series and 29 retrospective studies. Twenty-three studies used 5-Fu during bleb needling, while 17 used MMC. Three studies directly compared the efficacy of these antimetabolites [19, 26, 29]. The weighted mean age for all the cases was 63.3 years. The average duration of follow-up after bleb needling ranged from 6 to 60 months.  Table 1 summarizes the characteristics of the selected studies. Detailed technique of needling in each article was presented in Supplementary Table 1. Specifically, most of the included studies presented the data of former antimetabolites application of trabeculectomy.

### 3.3. Bias Assessment

**Figure 2:**
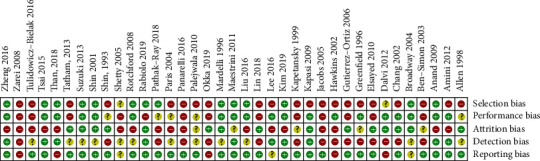
Summary of risk of bias assessment for each selected case.

We evaluated the risk of bias of all included articles.  Figure 2 displays a summary of bias in each domain with a graph presented in Supplementary Figure 1. The potential existence of publication bias was assessed across the studies by funnel plots in the analysis of IOP and medication changes after bleb needling. No significant asymmetry was found in the test (*p*=0.252,  0.114,  0.150,  0.063 and 0.715; for IOP reduction at six months, one year, two years, and last visit, antiglaucomatous medication reduction at last visit, resp.) (Supplementary Figures 2–6).

### 3.4. IOP Control

**Figure 3:**
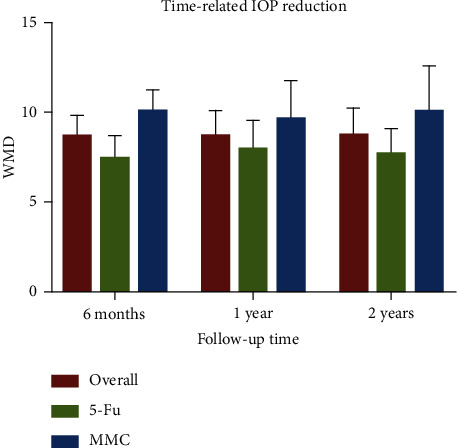
Column graph for time-related IOP reduction: weighted mean difference (WMD) of reduction at different timeline showed stable effects for bleb needling with 5-Fu and MMC.

**Figure 4:**
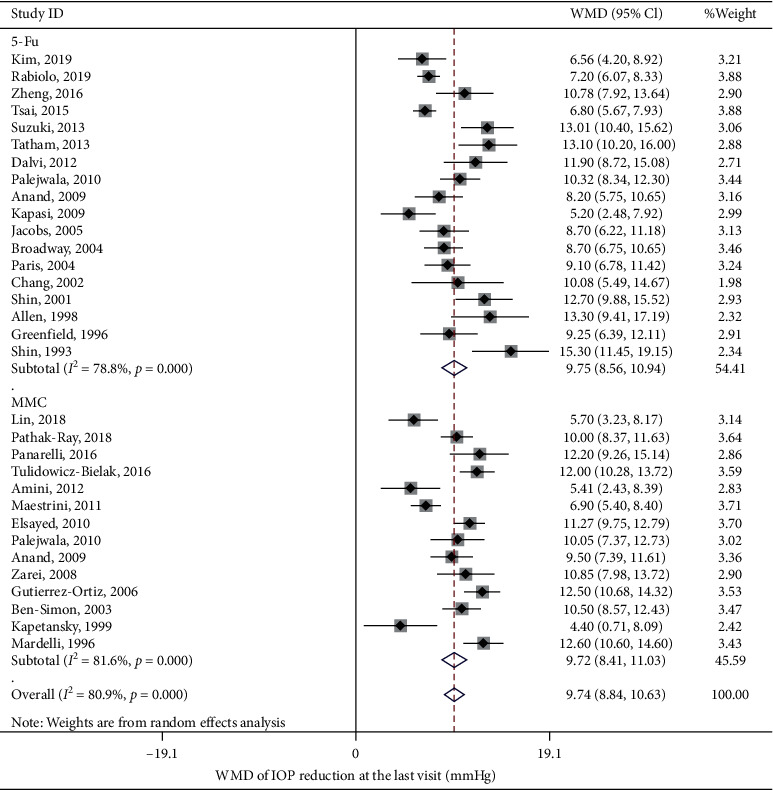
Weighted mean difference (WMD) of reduction in intraocular pressure (IOP) from baseline to the last visit. Subgroup analysis displayed the MMC and 5-Fu was used as an augmentation in bleb needling surgery.

**Figure 5:**
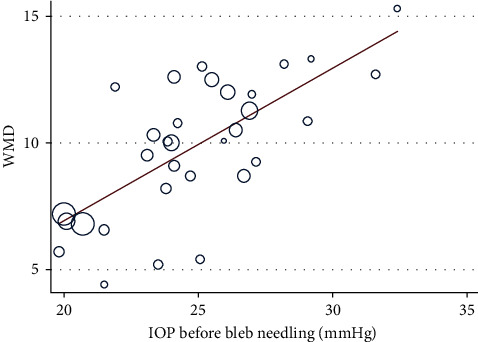
Metaregression for IOP before bleb needling: metaregression revealed that IOP before bleb needling significantly contributed to the heterogeneity of WMD of IOP reduction at last visit (*p* < 0.001).

IOP reduction levels at six months, one year, two years, and last visit after bleb needling were analyzed. Data at one year were regarded as last-visit data for the studies with loss of >50% cases at the last visit [12, 15–17]. Combined analysis with a random-effect model showed a significant reduction in IOP at a different timeline (*p* < 0.001). It decreased by a mean of 8.66 mmHg (95% CI [7.46, 9.85]; *I*^2^ = 78.4%; 12 studies) at six months, 8.69 mmHg (95% CI [7.25, 10.12]; *I*^2^ = 85.7%; 13 studies) at one year, 8.73 mmHg (95% CI [7.21, 10.25]; *I*^2^ = 85.1%; 11 studies) at two years (Supplementary Figures 7–9, Figure 3), and 9.74 mmHg (95% CI [8.85, 10.63]; *I*^2^ = 80.9%; 30 studies) at the last visit. Subgroup analysis was applied to explore sources of heterogeneity among the studies. Typically, bleb needling brought about 9.72 mmHg (95% CI [8.41, 11.03]; *I*^2^ = 81.6%) reduction with MMC injection at the last visit and 9.75 mmHg (95% CI [8.56, 10.94]; *I*^2^ = 79.9%) with 5-Fu injection. With 95% CI from baseline to the last visit, WMD was presented as a forest plot (Figure 4). Of note, most of the strata in the subgroup showed high heterogeneity (Supplementary Table 2). Sensitivity analysis revealed that the estimate of WMD was stable (Supplementary Figure 11). Further metaregression revealed that IOP before bleb needling contributed significantly to the heterogeneity of WMD of IOP reduction at last visit (*p* < 0.001), revealing a trend that higher baseline IOP correlated with a greater IOP reduction results (Figure 5, Supplementary Table 3). To be specific, it explained 64.9% of the between-study variance.

### 3.5. Estimate for the Success Rate

Criteria of success varied among studies. In reviewing the studies, the most accepted definition of complete success was IOP < 21 mmHg or reduction of 20% from baseline without any antiglaucomatous medication [2, 7, 9, 11, 13, 15, 19–22, 30, 31, 36, 38], while qualified success referred to the same IOP level with topical antiglaucomatous medication [7, 9, 11, 12, 15, 19–22, 30, 31, 36, 38]. Those with criteria stricter than the previously mentioned definition were considered eligible for the pooled analysis of success rate. In a random-effect model, the overall estimate of complete success rate for bleb needling at the last visit was 45.9% (95% CI [39.0%, 53.0%]; *I*^2^ = 84.1%; 22 studies), while qualified success rate was 70.4% (95% CI [63.5%, 77.0%]; *I*^2^ = 88.1%; 26 studies). Specifically, MMC injection subgroup reached 48.6% (95% CI [35.0%, 62.2%]) in complete success rate and 70.2% (95% CI [60.0%, 79.7%]) in qualified success rate, while 5-Fu subgroup was 44.3% (95% CI [36.3%, 52.4%]) and 70.7% (95% CI [60.9%, 79.6%]).

### 3.6. Antiglaucomatous Medication Reduction

Twenty studies had a record of antiglaucomatous medication usage before and after bleb needling. The postoperative number of medications was significantly fewer than the preoperative number of medications by 0.80 (95% CI [0.52, 1.09]; *p* < 0.001; Supplementary Figure 10).

### 3.7. Bleb Morphology

Needling will alter the morphology of failed bleb, though data are presented in limited studies [13, 17, 29, 35]. Flat blebs [13, 17, 35] and scared blebs [29], as the primary form of bleb characteristic in the studies, significantly shifted into diffuse blebs after needling [13, 35]. Four studies [11, 23, 30, 40] had focused on encapsulated bleb while one study [25] shed light on flat filtering bleb. However, no quantitative descriptions of morphology changes were presented in these studies.

As far as the relationship between bleb morphology and surgical outcomes was concerned, only seven studies were involved [2, 7, 8, 13, 17, 20, 35]. Based on different descriptions for bleb characteristics, the conclusion varied among studies. Lee et al. [20] claimed that greater central bleb extension and more elevated height were associated with a higher chance of success. Than et al. [13] showed that injected bleb appearance before needling was a significant indicator of success. Rotchford and King [2] considered bleb morphology a modifier rather than a risk factor for surgical outcomes. They displayed that when the bleb was injected or microcystic, bleb elevation at the time of needling correlated with longer survival time. However, IOP reduction had no significant difference in comparison of flat blebs and other types before needling [7, 8, 17]. While under a definition of postneedling IOP < 22 mmHg, or 30% reduction of IOP, the variation of success rate between bleb types did not reach statistical significance [35]. The predictive value for bleb morphology remained controversial.

### 3.8. Complications

A total of 29 studies, including 1661 cases, reported the occurrence of complications. We ran the meta-analysis based on these studies. An estimate of the incidence rate of complication throughout the whole course of study following bleb needling is presented in Table 2. Within all the complications, conjunctival haemorrhage (5.7%, 95% CI [2.5%, 10.1%]), hyphema (5.5%, 95% CI [3.0%, 8.7%]), and bleb leakage (5.0%, 95% CI [3.2%, 7.3%]) were the most commonly reported and had the highest estimate of incidence. Most of them can be resolved by conservative treatment or after observation. According to our estimation, complications that appeared after MMC injection might be slightly more than that of 5-Fu injection, especially in conjunctival haemorrhage (10.8% vs. 2.7%). Besides, some rare complications were listed as they were not suitable for aggregation (events/cases): vitreous haemorrhage (4%, 5/125) [25], cystoid macular oedema (2.4%, 4/266) [2, 10, 33], bullous keratopathy (1.7%, 5/290) [11, 17, 29, 31, 39], corneal endothelial decompensation (1.5%, 5/330) [2, 10, 14, 29], suprachoroidal haemorrhage (1.4%, 6/422) [2, 4, 17, 26, 29, 41], endophthalmitis (1.4%, 3/220) [10, 41], hypotony maculopathy (1.3%, 2/157) [10], and blebitis (0.6%, 1/157) [10]. The variance of incidence among studies was probably caused by the setting of different trials. Those with a longer duration of follow-up [15, 18, 29] and larger sample size [10, 17, 25, 35] reported relatively more complications. Typically, Maestrini et al. [25] reported a maximum of 173 cases of postoperative complications in a mean follow-up of 20.8 months for 125 eyes. Cataract was reported in four studies, but its correlation with bleb needling was uncertain [10, 14, 25, 33].

### 3.9. Possible Risk Factors for Surgical Outcome

Potential risk factors for the failure of bleb needling had been studied among 11 studies, mainly focusing on the number of needling, IOP, and time interval between needling and trabeculectomy [2, 10, 12–14, 20, 21, 29, 35, 37, 39].

Five studies concluded that the total number of needling was an independent risk factor [2, 12–14, 21]. In a long-term study, both median survival time and two-year success rate were significantly reduced for those who underwent more than one needling [2]. One study had calculated that the hazard ratio (HR) was 16.13 (95% CI, [1.22, 211.71]) [14]. However, given that this particular research was based on data with a wide range of distribution and relatively small sample size, the reliability had been lowered. HR conducted from studies with a larger sample size had a range between 1.35 [21] and 2.25 [12].

IOP was another potential risk factor in surgery failure. IOP drop immediately after the surgery [29], typically with the cut-off value of 10 mmHg [2, 13, 39], was a predictor for success. In comparison, among the eleven studies analyzed risk factors, only two studies showed that higher IOP before bleb needling was slightly more likely to cause a worse outcome [10, 21].

Timing selection for bleb needling after trabeculectomy was considered significant for surgical outcomes in two studies [2, 20]. Lee et al. [20] claimed that timing was the only significant risk factor in a Cox regression model. However, in another study, within three months after trabeculectomy, needling bleb can survive up to 12 months (HR = 2.2) but did not reach a significant outcome in the long-term [2].

## 4. Discussion

### 4.1. Key Findings

We performed a meta-analysis of 37 studies that included patients who underwent bleb needling with subconjunctival injection of antimetabolites after trabeculectomy failure. Our results showed that the bleb needling significantly lowered the IOP and reduced the number of antiglaucomatous medications. The overall effects of bleb needling with antimetabolites at the last visit revealed a reduction in IOP of 9.74 mmHg, 45.9% for complete success rate, and 70.4% for qualified success rate. Despite similar efficacy profiles for MMC and 5-Fu, MMC showed higher estimate rates of complications. Baseline IOP was found to be a significant source of heterogeneity between the studies and had a positive correlation with the IOP reduction level.

### 4.2. Efficacy and Possible Rationale

Most of the studies we included evaluated the efficacy by success rate. However, as the definition of success or failure varied among studies, we additionally selected IOP value as a primary outcome because of its objectivity. Specifically, Paris et al. [34] and Kapetansky and Kapetansky [8] concentrated on IOP alterations rather than success rate. The reduction of IOP value was significant after bleb needling but revealed no apparent difference between the antimetabolites [19, 26, 29], which correlated with our subgroup analysis results. As antimetabolites were applied to keep the wound from fibrosis, a similar effect on IOP reduction further validated the efficacy of bleb needling itself. Although long-term studies had proved that the impact could sustain for more than three years but gradually decrease in success rate with time [13, 15, 24, 29], an overall analysis of the IOP reduction level had shown a stable trend in more than six months in our results. Therefore, we considered that the present meta-analysis provides generalizable information for bleb needling in clinical settings.

Our results had revealed that MMC application was slightly better than 5-Fu in complete success rate but with higher complication rates, which corresponded to the potency of these medications. Direct comparison of MMC and 5-Fu application revealed that MMC outweighed 5-Fu in complete and qualified success rates with no significantly different complication rates [19, 29]. One retrospective study claimed no apparent difference between the two groups [26]. A possible explanation was that the modified criteria of success rate depending on the reduction rate instead of the absolute value of IOP might influence the results [26]. When considering the mechanism, antimetabolites modulate wound healing based on different targets. Typically, 5-Fu was specific for fibroblasts, while MMC inhibits both fibroblasts and endothelial cells [45]. Given this mechanism, MMC exerts a more potent and durable effect than 5-Fu and requires fewer doses during clinical practice.

As for secondary outcomes, the reduction of IOP at six months, one year, and two years remained stable in our pooled results, indicating a long-lasting effect of IOP control. However, only 5 of 37 included studies had a mean follow-up of over three years [13, 18, 24, 29, 39]. As the results were not sufficient for aggregation, estimates for the long-term effects of bleb needling were less robust in our meta-analysis.

### 4.3. Safety Profile

With regard to safety, our estimates of procedure-related adverse events were evaluated as overall rates among all the studies. Subgroup analysis revealed that MMC might have higher complication rates than 5-Fu, which was also relevant to the higher antifibrosis power and longer duration of MMC [45]. A direct comparison between MMC and 5-Fu showed no significant difference in the complication in an RCT [19]. Possible reasons may include the avoidance of insertion under the scleral flap or into the anterior chamber, which significantly reduced the incidence of complications for both medications. Concerning the statistics of specific studies, two studies classified the complications by time, among which hyphema [25] and corneal punctate epitheliopathy [10], respectively, had the highest rate during the early stage. Differences might originate from time selection for bleb needling between the studies, which was related to the scar formation, and the antifibrosis power of adjunctive antimetabolites [45].

### 4.4. Risk Factors

The total number of needling procedures revealed a significant influence on surgical outcomes among studies [2, 12–14, 21]. A possible explanation was that more attempts for needling in an individual patient represented a worse control of IOP after the initial procedure [2, 12]. Such a repetition of needling added the likelihood of poor prognosis. Specifically, for target IOP control, the number of needling tended to outweigh other factors with stricter criteria [13].

The predictive value of bleb morphology for surgical outcomes remained unclear [2, 7, 8, 13, 17, 20, 35]. Comparing the researches, the description of blebs varied greatly. A rough classification of bleb [7, 8, 13, 17] was more commonly used during evaluation, but its potential correlation with needling outcomes was weakened as it may ignore the severity of failed blebs. Though Than et al. [13] indicated the association of injected bleb and surgical success, the credibility was relatively low due to the subjective assessment for bleb morphology. A commonly-used Moorfields Bleb Grading System (MBGS) for bleb was therefore applied to evaluate the appearance [20]. Final success correlated with central bleb extension and height in this article. With this method, bleb morphology was comparable within different studies, yet it was not applied to the assessment for needling in most of the studies. Therefore, the relationship between bleb and needling outcomes remained to be explored.

As IOP and time selection were also considered predictive of surgical outcomes in different studies, risk factors for surgical failure defined as uncontrolled IOP did not reach a consensus among studies [2, 10, 12–14, 20, 21, 29, 35, 37, 39].

### 4.5. Heterogeneity

Heterogeneity was a major concern for our meta-analysis. Many had mentioned that the studies differ in design, sample sizes, follow-up periods, criterion for success, and so on [10, 13, 18], which made it difficult for comparison. Subgroup analysis in this study assessed the heterogeneity across the domains mentioned previously. Typically, regarding the bleb morphology before needling, a subgroup of three studies focusing on encapsulated bleb had lower heterogeneity than those not defined [23, 30, 40]. Considering the influence of a single study, Shin et al. [9] reported a maximum of 15.3 mmHg of mean IOP reduction, and Kapetansky and Kapetansky [8] reported a minimum of 4.4 mmHg. Nevertheless, sensitivity analysis revealed that exclusions of any single study did not alter the pooled results, possibly due to their small sample size, which added robustness to our main findings. Further metaregression displayed a positive linear trend between IOP before bleb needling and WMD, strongly indicating a primary source of heterogeneity. After adjusting, the heterogeneity level turned into moderate for the meta-analysis results. Given that the variance of study settings was high, we displayed the surgical details for bleb needling in each study. Of note, differences in technique included the site of puncture, distance from the scleral flap, anaesthesia, concentration of antimetabolites, entry beneath the scleral flap, steroid application, and so on. Currently, limited studies had focused on these variables in bleb needling. We reasoned that the high heterogeneity also possibly originated from glaucoma subtype, proficiency of the ophthalmologists, bleb morphology, limbus/fornix based trabeculectomy, phakic status, antimetabolites application, or dosage of antimetabolites, which were hard to evaluate within studies.

### 4.6. Strengths and Limitations

As far as we know, limited systemic reviews had focused on bleb needling [6]. The study including 25 cases with encapsulated blebs found similar efficacy for bleb needling and medical treatment but claimed the priority of the latter [46]. The conclusion was drawn based on small sample sizes and under the circumstances of less widely application of antimetabolites in that era. However, with the introduction of antimetabolites, bleb needling had become a more common procedure after trabeculectomy failure. Moreover, bleb needling was not limited to encapsulation in real-world settings. A recent RCT included 40 eyes with encapsulated blebs found that bleb needling with antimetabolites had a lower mean IOP at 12 months [23]. Another long-term outcome revealed that needling with 5-Fu for failing blebs had similar IOP control with those who had not undergone needling [24]. However, efficient as it was, bleb needling had displayed varied results due to diverse settings in a series of studies. For glaucoma specialists, the predictability of IOP reduction value and complication rates was crucial for treatment options. Our results were more practical with the inclusion of a larger sample and updated data for bleb needling with antimetabolites. Therefore, it can serve as a reference for treatment selection based on the estimations.

Several limitations of our study still need to be acknowledged. First, since a majority of studies were retrospective and noncomparative, variations in study design, methodology, and patient characteristics inevitably introduced relatively high heterogeneity, which we had discussed previously. Potential bias may lower the quality of our results. Second, the direct comparison of the antimetabolites was insufficient due to the original article setting we included. Although we observed the trends that MMC had slightly better IOP control than 5-Fu, the significance could not be told based on our pooled analysis. Third, criteria of success were modified in different studies based on their data. Those with a higher cut-off of IOP value and acceptance of repeating needling may potentially gain a higher success rate. As we chose a less strict definition, underestimation of success rate may be introduced during the pooled analysis. Moreover, neither short-term (<6 months) nor long-term (>2 years) efficacy of bleb needling was presented due to lack of data. Typically, owing to the probable loss of follow-up, long-term data with high quality may still be needed. Further direction for bleb needling researches may include long-term prospective study comparing the efficacy and safety for different adjuvant. The control group should be selected tactfully to increase the reliability of clinical judgment and treatment options in future studies.

## 5. Conclusion

In conclusion, bleb needling with antimetabolites could be considered an effective and safe procedure after trabeculectomy failure. After the process, patients will gain ideal IOP control and reduce antiglaucomatous medications for at least six months with 5-Fu or MMC. Meanwhile, the overall estimates for complications were relatively low in the whole process. An increasing number of needling was the leading risk factor for needling failure.

## Figures and Tables

**Table 1:** Baseline characteristics of included studies.

Author, year	Design	Number of eyes	Mean age (SD) (years)	Mean follow-up (SD) (months)	Diagnosis for study eyes (numbers (%))	Phakic status for study eyes (numbers (%))	Medication	Country/region
Rabiolo et al., 2019 [10]	Noncomparative, retrospective	157	65.8 (13.9)	25 (30.37)	POAG: 85 (54.1) PACG: 11 (7.0) Secondary glaucoma: 61 (38.9)	Pseudo: 140 (89.2); phakic: 16 (10.2); aphakic: 1 (0.6)	5-Fu	Italy
Okka et al., 2019 [11]	Noncomparative, retrospective	16	46 (18.2)	12.22 (6.08)	POAG: 9 (56.3) Secondary glaucoma: 7 (44)	N/A	5-Fu	Turkey
Kim et al., 2019 [12]	Comparative, retrospective	35	58.5 (13.1)	>6 (N/A)	POAG: 21 (60.0) PACG: 3 (8.6) Secondary glaucoma: 11 (31.4)	N/A	5-Fu	South Korea
Than et al., 2018 [13]	Noncomparative, retrospective	96	67 (15)	37.08 (21)	POAG: 70 (72.9) PACG: 6 (6.25) Secondary glaucoma: 18 (18.75) N/A: 2 (2.1)	Pseudo: 24 (25); phakic: 72 (75)	5-Fu	UK
Pathak-Ray and Choudhari, 2018 [14]	Noncomparative, prospective	39	59 (14.25)	20 (11)	POAG: 9 (23.1) PACG: 21 (53.8) Secondary glaucoma: 9 (23.1)	Pseudo: 26 (66.7); phakic: 13 (33.3)	MMC	India
Lin et al., 2018 [15]	Noncomparative, retrospective	44	73.7 (N/A)	58.7 (N/A)	POAG: 29 (65.9) PACG: 7 (15.9) Secondary glaucoma: 8 (18.2)	Pseudo: 28 (63.6); phakic: 16 (36.4)	MMC	UK
Zheng et al., 2016 [16]	Noncomparative, retrospective	33	68.67 (10.38)	21.72 (18.72)	POAG: 21 (67.7) PACG: 2 (6.5) Secondary glaucoma: 8 (25.8)^#^	N/A	5-Fu	Australia
Tulidowicz-Bielak et al., 2016 [17]	Noncomparative, retrospective	121	67.38 (13.76)	27.6 (N/A)	POAG: 121 (100)	Single needling: pseudo: 62 (51.2); phakic: 38 (31.4); multiple needling: unknown 21 (17.4)	MMC	Poland
Panarelli et al., 2016 [18]	Noncomparative, retrospective	27	69.1 (13.1)	54.2 (30.4)	POAG: 18 (66.7) PACG: 2 (7.4) Secondary glaucoma: 7 (25.9)	Pseudo: 17 (63.0); phakic: 9 (33.3); aphakic: 1 (3.7)	MMC	USA
Liu et al., 2016 [19]	RCT	75	48.5 (10.4)	12 (N/A)	MMC : POAG: 14 (35.0) PACG: 23 (57.5) Juvenile glaucoma: 3 (7.5) 5-Fu : POAG: 12 (34.3) PACG: 20 (57.1) Juvenile glaucoma: 1 (2.9) Secondary glaucoma: 2 (5.7)	N/A	MMC/5-Fu	China
Lee et al., 2016 [20]	Comparative, retrospective	41	52.1 (15.8)	22.7 (9.4)	POAG: 8 (19.5) PACG: 5 (12.2) Secondary glaucoma: 28 (68.3)	Pseudo: 19 (46.3); phakic: 21 (51.2); aphakic: 1 (2.4)	5-Fu	China
Tsai et al., 2015 [21]	Comparative, retrospective	227	66.9 (11.2)	>6 (N/A)	POAG: 157 (69.2) PACG: 70 (30.8)	N/A	5-Fu	Singapore
Tatham et al., 2013 [22]	Comparative, retrospective	34	67.88 (N/A)	25.2 (1.2)	POAG: 21 (61.8) PACG: 5 (14.7) Secondary glaucoma: 8 (23.5)	Pseudo: 17 (50.0); phakic: 17 (50.0)	5-Fu	UK
Suzuki and Susanna-Jr, 2013 [23]	RCT	20	57.3 (15.21)	12 (N/A)	POAG: 17 (85.0) Congenital glaucoma: 1 (5.0) Secondary glaucoma: 2 (10.0)	N/A	5-Fu	Brazil
Dalvi et al., 2012 [24]	Comparative, retrospective	40	67.83 (12.02)	60 (N/A)	POAG: 28 (70.0) PACG: 6 (15.0) Secondary glaucoma: 6 (15.0)	Pseudo: 20 (50.0); phakic: 20 (50.0)	5-Fu	Canada
Amini et al., 2012 [7]	Noncomparative, retrospective	27	56.5 (16.2)	20.31 (15.63)	POAG: 7 (26.0) PACG: 4 (14.8) Juvenile glaucoma: 4 (14.8) Secondary glaucoma: 11 (40.7) Developmental glaucoma: 1 (3.7)	N/A	MMC	Iran
Maestrini et al., 2011 [25]	Noncomparative, prospective	125	61.6 (18.8)	20.8 (12)	POAG: 97 (77.6) PACG: 10 (8.0) Congenital glaucoma: 6 (4.8) Secondary glaucoma: 12 (9.6)	Pseudo: 53 (42.4); phakic: 69 (55.2); aphakic: 3 (2.4)	MMC	Brazil
Palejwala et al., 2010 [26]	Comparative, retrospective	107	74.35 (11.95)	10.55 (11.01)	POAG: 81 (75.7) PACG: 3 (2.8) Secondary glaucoma: 23 (21.5)	Pseudo: 48 (44.9); phakic: 58 (54.2); aphakic: 1 (0.9)	MMC/5-Fu	USA
Elsayed and El-Raggal, 2010 [27]	Noncomparative, retrospective	30	7.3 (3.4)	9.23 (5.25)	Congenital glaucoma: 30 (100)	N/A	MMC	Egypt
Kapasi and Birt, 2009 [28]	Noncomparative, retrospective	37	71.2 (12.6)	24 (N/A)	N/A	N/A	5-Fu	Canada
Anand and Khan, 2009 [29]	Comparative, retrospective	98	73.5 (11.1)	53 (18.12)	5-Fu: POAG: 45 (88.2) PACG: 5 (9.8) Secondary glaucoma: 1 (2.0) MMC: POAG: 37 (841) PACG: 4 (9.0) Secondary glaucoma: 3 (6.9)^#^	MMC group: pseudo: 28 (62.2); phakic: 17 (37.8); 5-Fu group: pseudo: 30 (56.6); phakic: 23 (43.4)	MMC/5-Fu	UK
Zarei et al., 2008 [30]	Noncomparative, prospective	33	45.67 (22.41)	9.24 (5.27)	POAG: 9 (27.3) PACG: 4 (12.1) Congenital glaucoma: 6 (18.2) Juvenile glaucoma: 4 (12.1) Developmental glaucoma: 1 (2.7) Secondary glaucoma: 9 (27.3)	N/A	MMC	Iran
Rotchford and King, 2008 [2]	Noncomparative, prospective	81	70 (N/A)	40.27 (N/A)	POAG: 56 (69.1) PACG: 8 (9.9) secondary glaucoma: 17 (21.0)	Pseudo: 27 (33.3); phakic: 54 (66.7)	5-Fu	UK
Gutierrez-Ortiz et al., 2006 [31]	Noncomparative, prospective	34	65.9 (8.3)	14.2 (9.8)	POAG: 23 (67.6) PACG: 5 (14.7) Juvenile glaucoma: 1 (2.9) Secondary glaucoma: 5 (14.7)	N/A	MMC	Spain
Shetty et al., 2005 [32]	Noncomparative, retrospective	44	72.9 (10.4)	>12 (NA)	POAG: 35 (79.5) PACG: 4 (9.1) Secondary glaucoma: 5 (11.4)	Pseudo: 31 (70.5); phakic: 12 (27.3); aphakic: 1 (2.3)	MMC	USA
Jacobs et al., 2005 [33]	Noncomparative, retrospective	28	61 (N/A)	14 (N/A)	N/A	N/A	5-Fu	Belgium
Paris et al., 2004 [34]	Noncomparative, retrospective	36	N/A	6 (N/A)	POAG: 36 (100)	N/A	5-Fu	USA
Broadway et al., 2004 [35]	Noncomparative, retrospective	101	69 (11)	20.2 (NA)	POAG: 71 (70.2) PACG: 5 (5.0) Secondary glaucoma: 25 (24.8)	Pseudo: 16 (15.8); phakic: 12 (79.2); aphakic: 5 (5.0)	5-Fu	UK
Ben-Simon and Glovinsky, 2003 [36]	Noncomparative, retrospective	41	65 (17)	6 (N/A)	POAG: 26 (63.4) PACG: 2 (4.9) Congenital glaucoma: 2 (4.9) Secondary glaucoma: 11 (26.8)	Pseudo: 22 (53.7); phakic: 13 (31.7); aphakic: 6 (14.6)	MMC	Israel
Hawkins et al., 2002 [37]	Noncomparative, retrospective	49	70.14 (13.74)	19.9 (16.3)	POAG: 25 (58.1) PACG: 8 (18.6) Secondary glaucoma: 10 (23.3)^#^	Pseudo/aphakic: 32 (74.4); phakic: 11 (25.6)	5-Fu	USA
Chang and Hou, 2002 [38]	Noncomparative, retrospective	25	53.0 (13.2)	8.32 (6.61)	POAG: 4 (16.0) PACG: 4 (16.0) Secondary glaucoma: 17 (68.0)	Pseudo: 12 (48.0); phakic: 6 (24.0); aphakic: 7 (28.0)	5-Fu	China
Shin et al., 2001 [39]	Noncomparative, retrospective	64	72.1 (9.5)	44.2 (21.2)	POAG: 61 (95.3) PACG: 2 (3.1) Secondary glaucoma: 1 (1.6)	Pseudo: 10 (15.6); phakic: 51 (79.7); aphakic: 3 (4.7)	5-Fu	USA
Kapetansky and Kapetansky, 1999 [8]	Comparative, retrospective	30	N/A	>6 (N/A)	N/A	N/A	MMC	USA
Allen et al., 1998 [40]	Noncomparative, retrospective	32	63.9 (10.8)	10.7 (2.9)	POAG: 20 (62.5) PACG: 5 (15.6) Secondary glaucoma: 7 (21.9)	Pseudo: 6 (18.8); phakic: 24 (75.0); aphakic: 2 (6.2)	5-Fu	UK
Mardelli et al., 1996 [4]	Noncomparative, retrospective	62	72 (15)	9.9 (3.7)	POAG: 32 (51.6) PACG: 9 (14.5) Congenital glaucoma: 4 (6.5) Secondary glaucoma: 17 (27.4)	Pseudo: 42 (67.8); phakic: 17 (27.4); aphakic: 3 (4.8)	MMC	USA
Greenfield et al., 1996 [41]	Comparative, retrospective	63	72.3 (11.2)	13.1 (8.1)	POAG: 32 (50.8) PACG: 9 (14.3) Secondary glaucoma: 22 (34.9)	Pseudo: 27 (42.9); phakic: 36 (57.1)	MMC	USA
Shin et al., 1993 [9]	Comparative, retrospective	30	63.5 (14.5)	15.5 (5.2)	POAG: 22 (73.3) Secondary glaucoma: 8 (26.7)	Pseudo: 17 (56.7); phakic: 10 (33.3); aphakic: 3 (10.0)	5-Fu	USA

^*∗*^MMC: mitomycin C; PACG: primary angle-closure glaucoma; POAG: primary open-angle glaucoma; RCT: randomized-control trial; SD: standard deviation; 5-Fu: 5-fluorouracil. N/A meant that the data were not presented in the original article. ^*∗∗*^Secondary glaucoma included pseudoexfoliated glaucoma, uveitic glaucoma, pigmentary glaucoma, traumatic glaucoma, steroid-induced glaucoma, neovascular glaucoma, iridocorneal endothelial syndrome, and so on. Developmental glaucoma included Axenfeld-Reiger Syndrome, Sturge-Weber Syndrome, and so on. ^*#*^Number of patients was displayed.

**Table 2:** Estimates with 95% confidence interval for complication rates after bleb needling.

	Overall		5-Fu		MMC	
	Estimate (%)	95% CI	Estimate (%)	95% CI	Estimate (%)	95% CI
Conjunctival haemorrhage	5.7	[2.5%, 10.1%]	2.7	[1.1%, 4.9%]	10.8	[2.9%, 22.9%]
Hyphema	5.5	[3.0%, 8.7%]	3.9	[1.8%, 6.8%]	7.4	[3.0%, 13.6%]
Bleb leakage	5.0	[3.2%, 7.3%]	5.3	[2.9%, 8.4%]	4.8	[2.1%, 8.4%]
Hypotony	4.3	[2.9%, 6.0%]	3.6	[1.9%, 5.9%]	6.0	[3.0%, 8.0%]
Shallow anterior chamber	3.4	[1.6%, 5.8%]	1.7	[0.6%, 3.4%]	5.6	[1.9%, 11.0%]
Serous choroidal detachment	2.6	[1.2%, 4.5%]	1.5	[0.6%, 2.7%]	4.1	[1.3%, 8.5%]
Corneal punctuate epitheliopathy	2.4	[1.1%, 4.2%]	2.0	[0.4%, 4.7%]	2.9	[1.1%, 5.6%]
Choroidal effusion	1.3	[0.6%, 2.1%]	1.5	[0.7%, 2.7%]	1.0	[0.2%, 2.3%]

^*∗*^CI: confidence interval; MMC: mitomycin C; 5-Fu: 5-fluorouracil.

## Data Availability

The pooled analysis data used in this study are available from the corresponding author upon request.

## Supplementary Materials

Supplementary Materials The search strategy was included in the supplementary file. Supplementary Figure 1: the risk of bias graph for included studies. Supplementary Figures 2–6: the funnel plots for WMD of IOP reduction at the different timeline and changes in antiglaucomatous medications. Supplementary Figures 7–10: the forest plots for WMD of IOP reduction at the different timeline and changes in antiglaucomatous medications. Supplementary Figure 11: the sensitivity analysis for WMD of IOP reduction at the last visit. Supplementary Table 1: the detailed technique of bleb needling in each study. Supplementary Table 2: subgroup analysis results for WMD of IOP reduction at the last visit. Supplementary Table 3: metaregression results for WMD of IOP reduction at the last visit.
