# Supplementary material for: Safety and Efficacy of Bleb Needling with Antimetabolite after Trabeculectomy Failure in Glaucoma Patients: A Systemic Review and Meta-Analysis
Source: J Ophthalmol. 2020 Nov 30;2020:4310258. doi: 10.1155/2020/4310258 (PMC7722638; doi:10.1155/2020/4310258)
Supplement: Supplementary Materials — The search strategy was included in the supplementary file. Supplementary Figure 1: the risk of bias graph for included studies. Supplementary Figures 2–6: the funnel plots for WMD of IOP reduction at the different timeline and changes in antiglaucomatous medications. Supplementary Figures 7–10: the forest plots for WMD of IOP reduction at the different timeline and changes in antiglaucomatous medications. Supplementary Figure 11: the sensitivity analysis for WMD of IOP reduction at the last visit. Supplementary Table 1: the detailed technique of bleb needling in each study. Supplementary Table 2: subgroup analysis results for WMD of IOP reduction at the last visit. Supplementary Table 3: metaregression results for WMD of IOP reduction at the last visit. [file 4310258.f1.doc]

# Safety and Efficacy of Bleb Needling with Antimetabolite After Trabeculectomy Failure in Glaucoma Patients-A Systemic Review and Meta-analysis——Supplementary

Xuhao Chen^1, 2^, Lingge Suo^1, 2^, Ying Hong^1, 2^*, Chun Zhang^1, 2^

^1^Department of Ophthalmology, Peking University Third Hospital, Beijing, China

^2^Beijing Key Laboratory of Restoration of Damaged Ocular Nerve, Peking University Third hospital, Beijing, China

*Correspondence:

Ying Hong, MD

Department of Ophthalmology, Peking University Third Hospital

49 North Garden Road, Haidian District, Beijing 100191, P R China

Email: [drhongying@bjmu.edu.cn](mailto:drhongying@bjmu.edu.cn)

Catalogue

[Supplementary: Search strategy 2](#__RefHeading___Toc47559830)

[Supplementary Figure 1: Risk of bias graph 4](#__RefHeading___Toc47559831)

[Supplementary Figure 2: Funnel plots for WMD of IOP reduction at 6 months 5](#__RefHeading___Toc47559832)

[Supplementary Figure 3: Funnel plots for WMD of IOP reduction at 1 year 6](#__RefHeading___Toc47559833)

[Supplementary Figure 4: Funnel plots for WMD of IOP reduction at 2 years 7](#__RefHeading___Toc47559834)

[Supplementary Figure 5: Funnel plots for WMD of IOP reduction at the last visit 8](#__RefHeading___Toc47559835)

[Supplementary Figure 6: Funnel plots for WMD of antiglaucomatous medications reduction at the last visit 9](#__RefHeading___Toc47559836)

[Supplementary Figure 7: Forest plot for WMD of IOP reduction at 6 months 10](#__RefHeading___Toc47559837)

[Supplementary Figure 8: Forest plot for WMD of IOP reduction at 1 year 11](#__RefHeading___Toc47559838)

[Supplementary Figure 9: Forest plot for WMD of IOP reduction at 2 years 12](#__RefHeading___Toc47559839)

[Supplementary Figure 10: Forest plot for WMD of antiglaucomatous medication reduction at the last visit 13](#__RefHeading___Toc47559840)

[Supplementary Figure 11: Sensitivity analysis for WMD of IOP reduction at last visit 14](#__RefHeading___Toc47559841)

[Supplementary Table 1: Detailed technique of bleb needling for each study 15](#__RefHeading___Toc47559842)

[Supplementary Table 2: Subgroup analyses for WMD for IOP reduction at the last visit 19](#__RefHeading___Toc47559843)

[Supplementary Table 3: Meta-regression for WMD for IOP reduction at the last visit 20](#__RefHeading___Toc47559844)

# Supplementary: Search strategy

**PubMed**

(((((fail*) OR encapsulat*) OR needl*) OR revis*) OR reconstruct*) AND ((filtering bleb) OR ((blister[MeSH Terms]) OR bleb*)) AND (((trabeculectomy[MeSH Terms]) OR trabeculectomy) OR ((((filtering surgery[MeSH Terms]) OR filter*) OR filtrat*) OR drain*))

**Embase**

| No. | Query |
| --- | --- |
| #17 | #12 AND #16 |
| #16 | #11 OR #15 |
| #15 | #13 AND #14 |
| #14 | #6 OR #7 OR #8 OR #9 OR #10 |
| #13 | #4 OR #5 |
| #12 | #1 OR #2 OR #3 |
| #11 | 'bleb needling'/exp OR 'bleb needling' |
| #10 | 'revision surgery'/exp OR 'revision surgery' |
| #9 | 'reconstructive surgery'/exp OR 'reconstructive surgery' |
| #8 | 'encapsulated bleb'/exp OR 'encapsulated bleb' |
| #7 | 'failure'/exp OR 'failure' |
| #6 | 'needling'/exp OR 'needling' |
| #5 | 'blister'/exp OR blister OR 'bleb'/exp OR 'bleb' |
| #4 | 'filtering bleb'/exp OR 'filtering bleb' |
| #3 | filter* OR filtrat* OR drain* |
| #2 | 'filtering operation'/exp OR 'filtering operation' |
| #1 | 'trabeculectomy'/exp OR 'trabeculectomy' |

**Cochrane**

#1 (trabeculectomy):ti,ab,kw

#2 (filter* OR filtrat* OR drain*):ti,ab,kw

#3 MeSH descriptor: [Trabeculectomy] explode all trees

#4 MeSH descriptor: [Filtering Surgery] explode all trees

#5 #1 OR #2 OR #3 OR #4

#6 MeSH descriptor: [Blister] explode all trees

#7 (bleb):ti,ab,kw

#8 (filtering bleb):ti,ab,kw

#9 #6 OR #7 OR #8

#10 (fail* OR encapsulat*):ti,ab,kw

#11 (needl* OR revis* OR reconstruct*):ti,ab,kw

#12 #10 OR #11

#13 #5 AND #9 AND #12

**Clinicaltrail.gov**

trabeculectomy AND bleb needling

# Supplementary Figure 1: Risk of bias graph


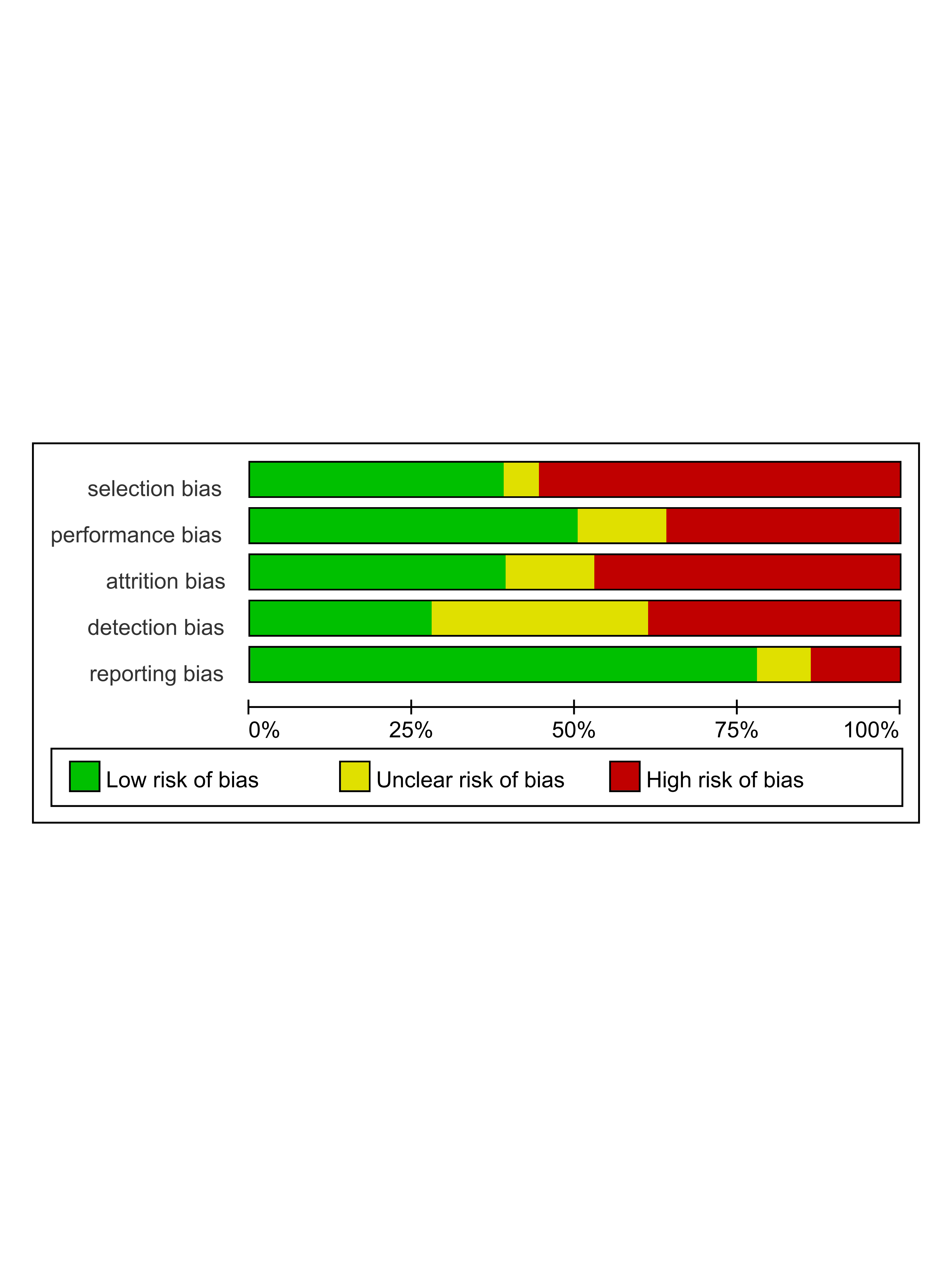


**Supplementary Figure 1** **Risk of bias graph**

# Supplementary Figure 2: Funnel plots for WMD of IOP reduction at 6 months


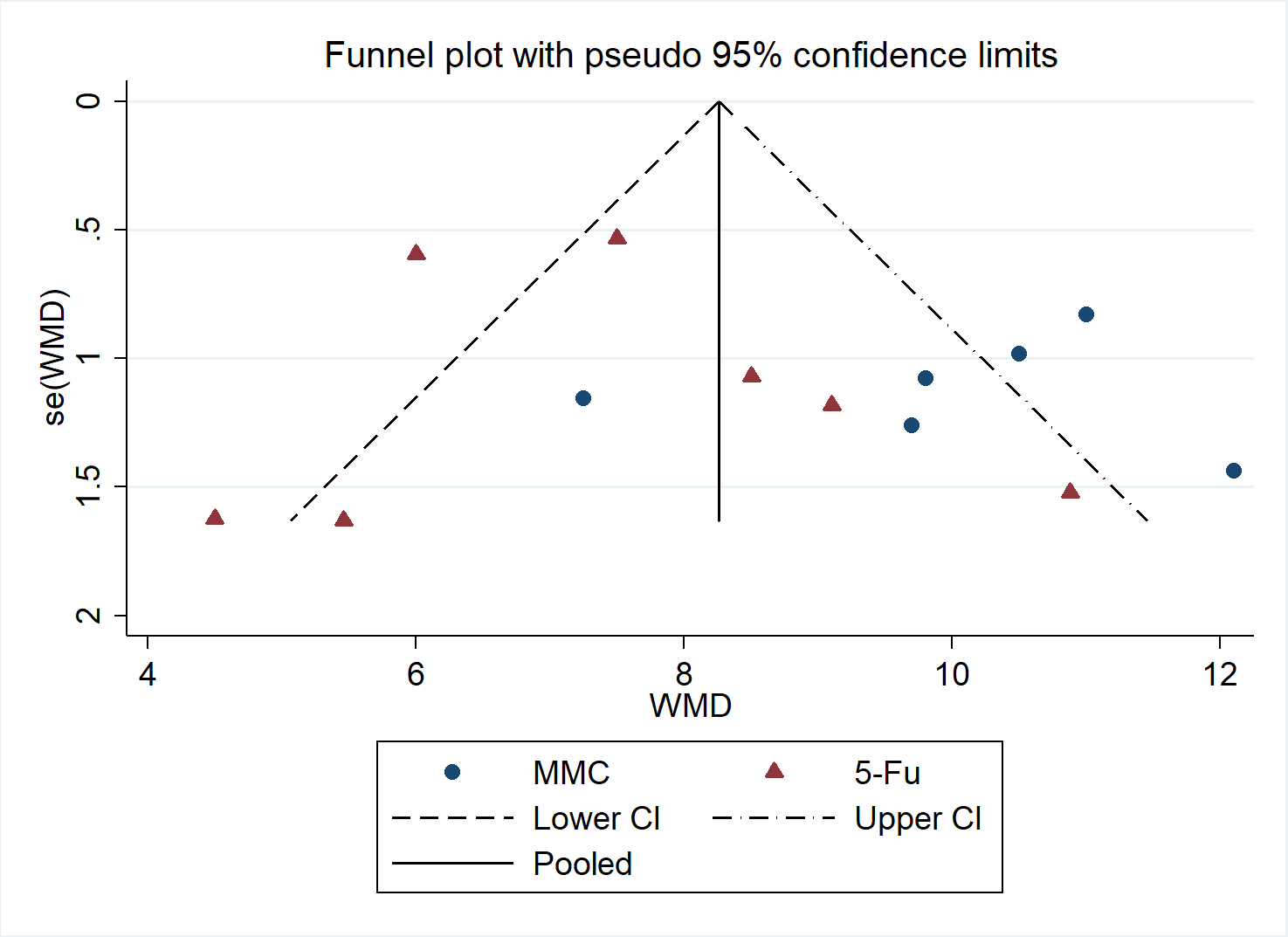


**Supplementary Figure 2** **Funnel plots for WMD of IOP reduction at 6 months**: Funnel plot of weighted mean difference (WMD) versus standard error (SE) of WMD for IOP before bleb needling and at 6 months after the procedure. (p=0.485)

# Supplementary Figure 3: Funnel plots for WMD of IOP reduction at 1 year


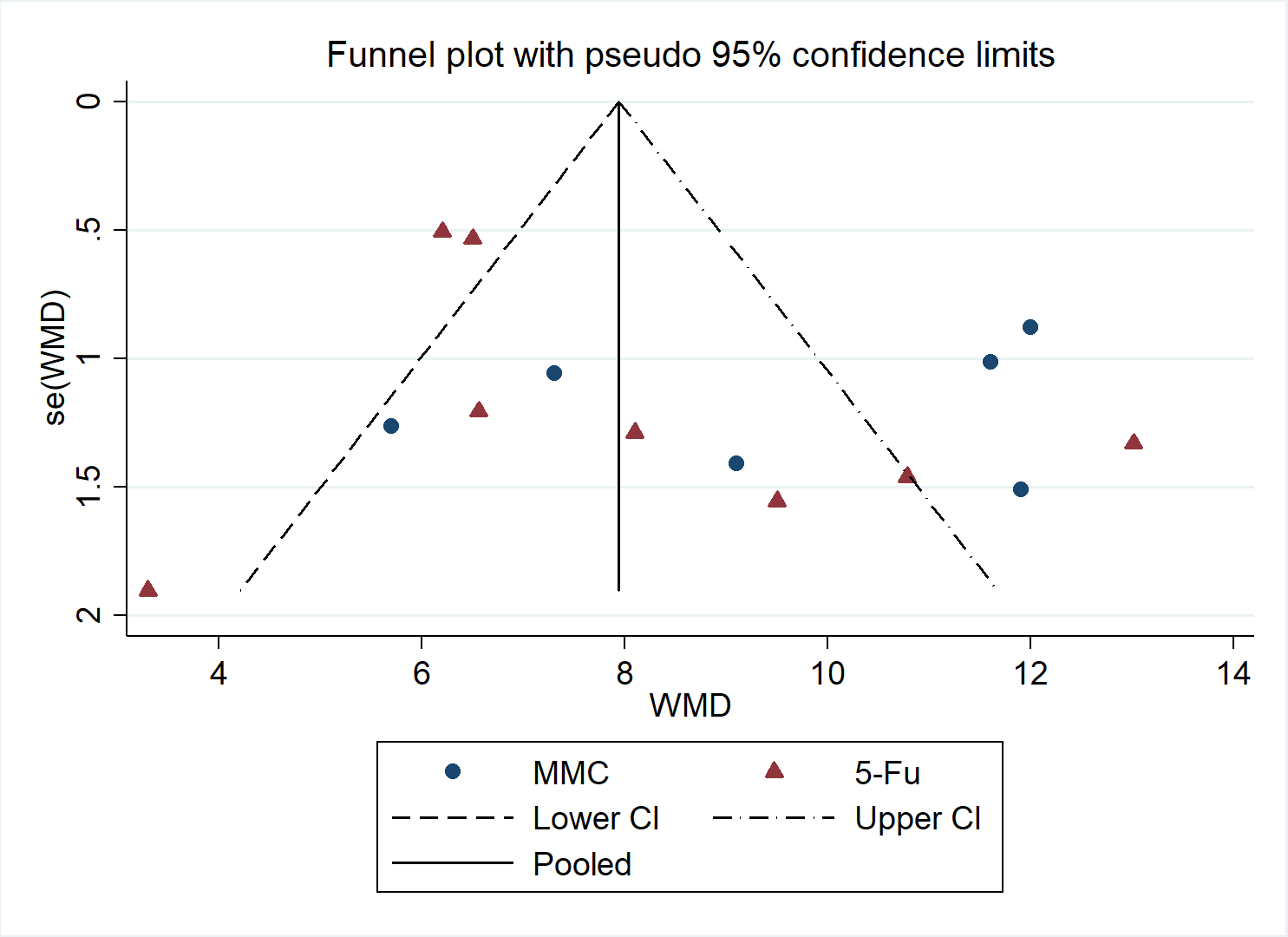


**Supplementary Figure 3** **Funnel plots for WMD of IOP reduction at 1 year**: Funnel plot of weighted mean difference (WMD) versus standard error (SE) of WMD for IOP before bleb needling and at 1 year after the procedure. (p=0.114)

# Supplementary Figure 4: Funnel plots for WMD of IOP reduction at 2 years


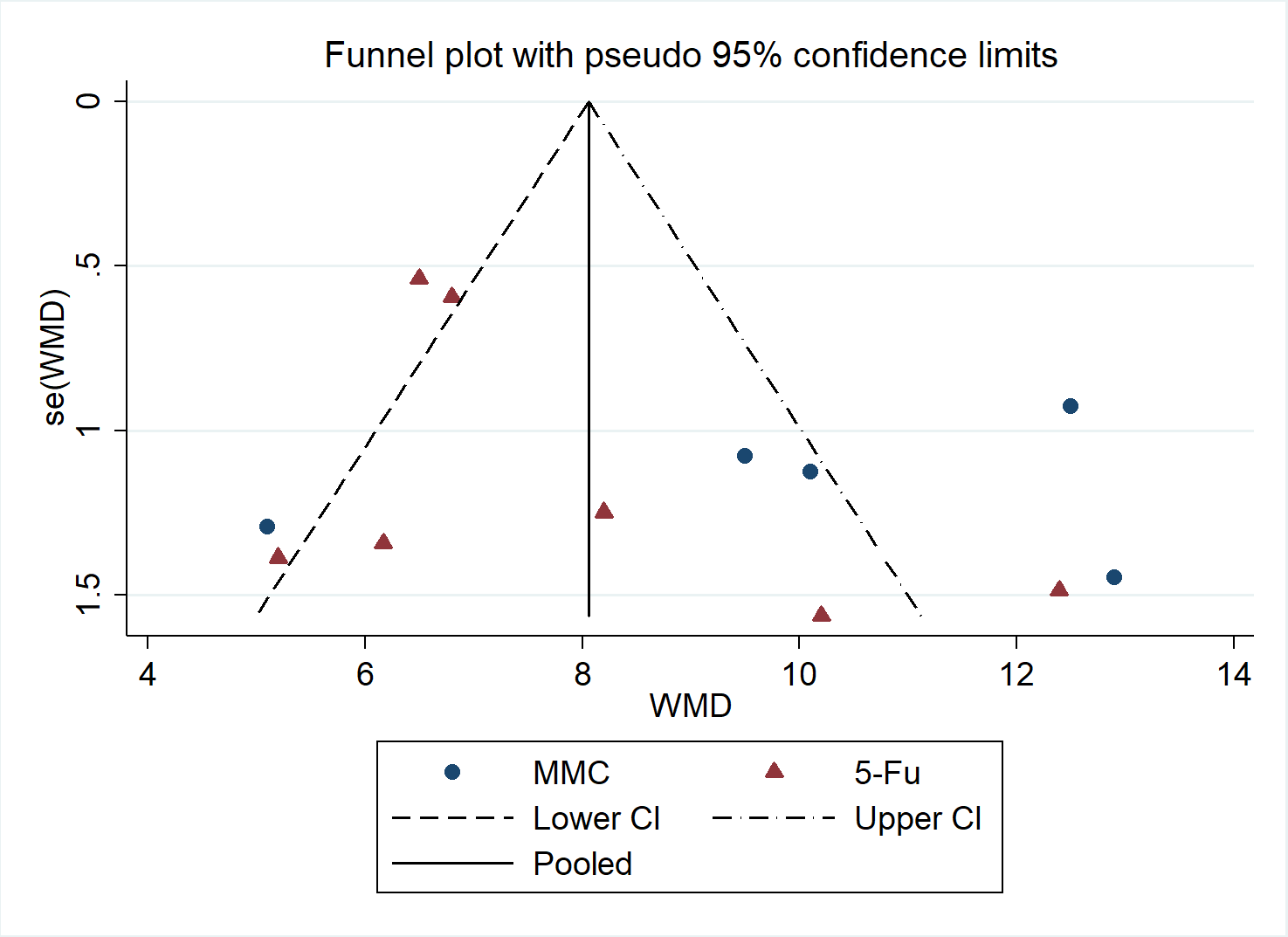


**Supplementary Figure 4** **Funnel plots for WMD of IOP reduction at 2 years**: Funnel plot of weighted mean difference (WMD) versus standard error (SE) of WMD for IOP before bleb needling and at 2 years after the procedure. (p=0.189)

# Supplementary Figure 5: Funnel plots for WMD of IOP reduction at the last visit


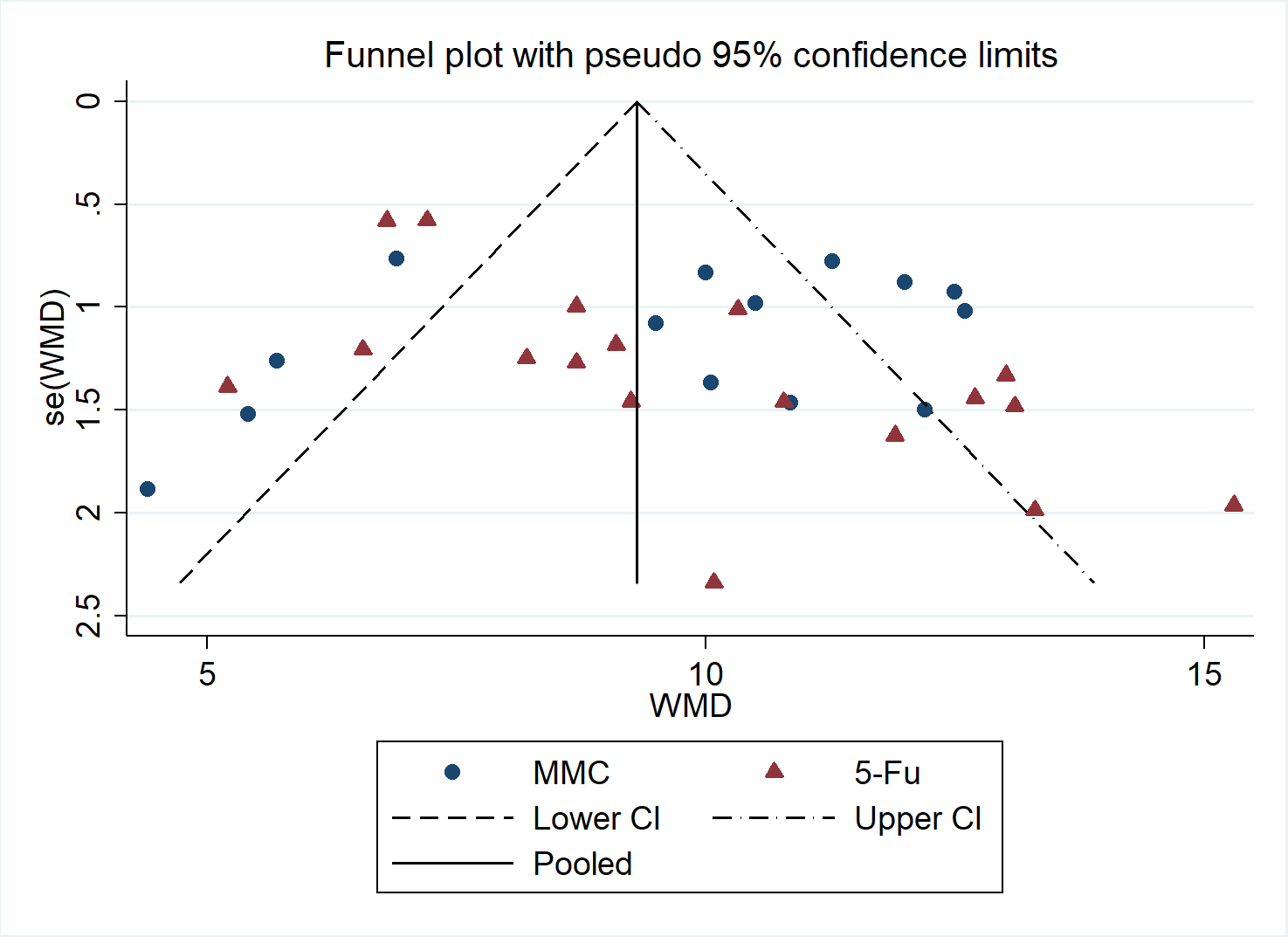


**Supplementary Figure 5** **Funnel plots for WMD of IOP reduction at the last visit**: Funnel plot of weighted mean difference (WMD) versus standard error (SE) of WMD for IOP before bleb needling and at last visit after the procedure. (p=0.063)

# Supplementary Figure 6: Funnel plots for WMD of antiglaucomatous medications reduction at the last visit


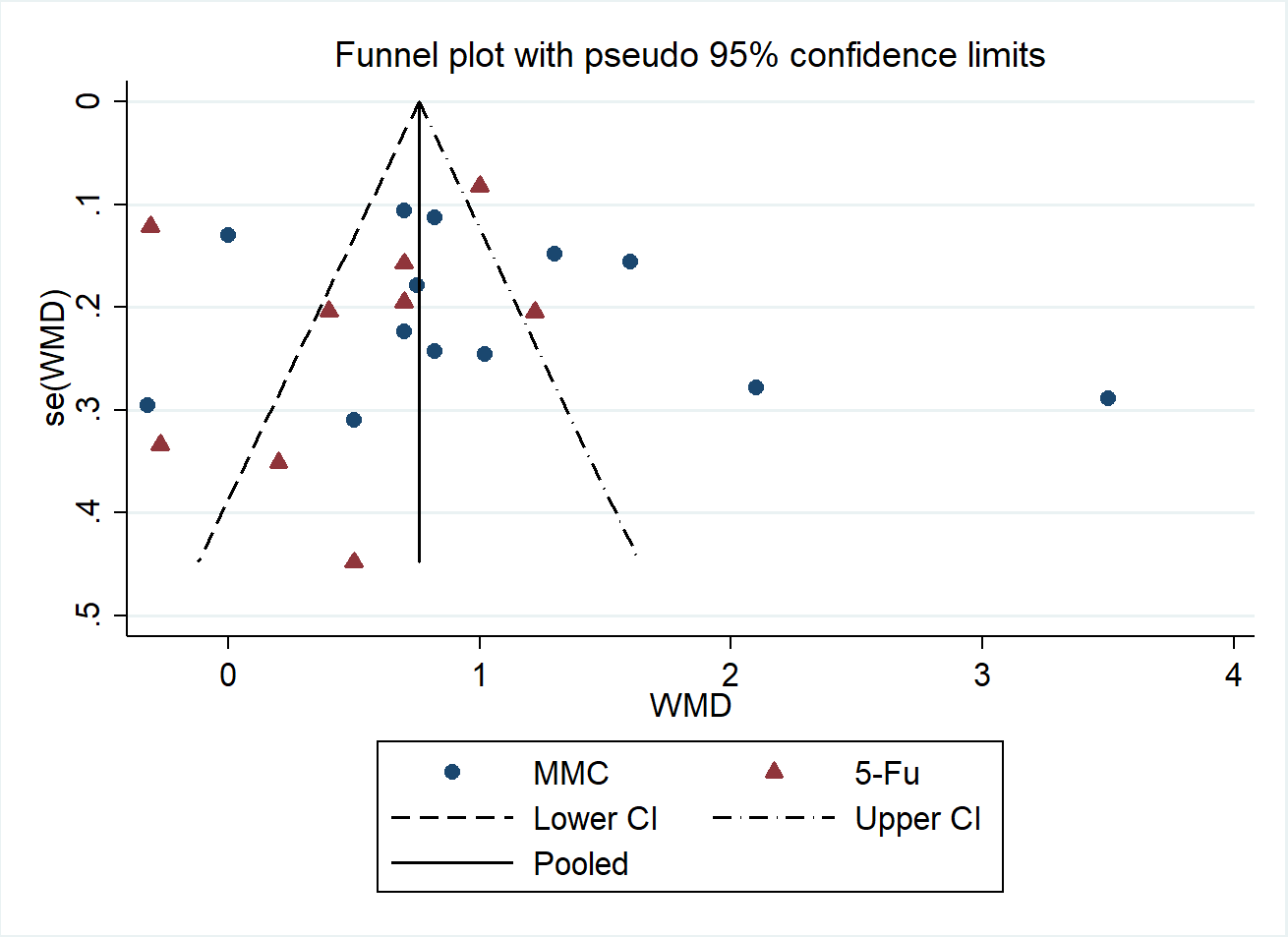


**Supplementary Figure 6** **Funnel plots for WMD of antiglaucomatous medications reduction at the last visit**: Funnel plot of weighted mean difference (WMD) versus standard error (SE) of WMD for topical medication before bleb needling and at last visit after the procedure. (p=0.715)

# Supplementary Figure 7: Forest plot for WMD of IOP reduction at 6 months


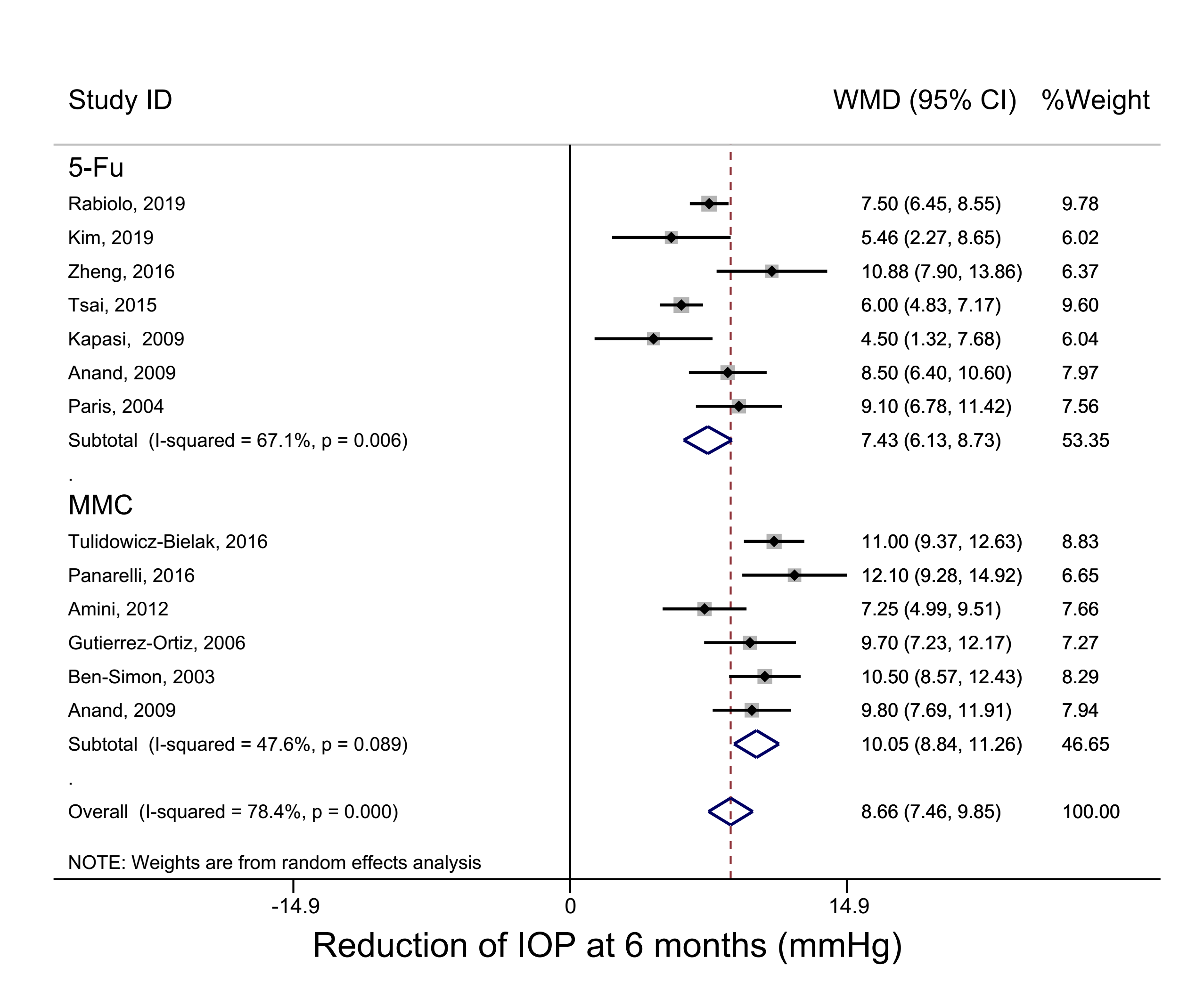


**Supplementary Figure 7 Forest plots for WMD of IOP reduction at 6 months**: Weighted mean difference (WMD) of reduction in intraocular pressure (IOP) from baseline to 6 months. Subgroup analysis displayed the MMC and 5-Fu used as an adjuvant in bleb needling surgery.

# Supplementary Figure 8: Forest plot for WMD of IOP reduction at 1 year


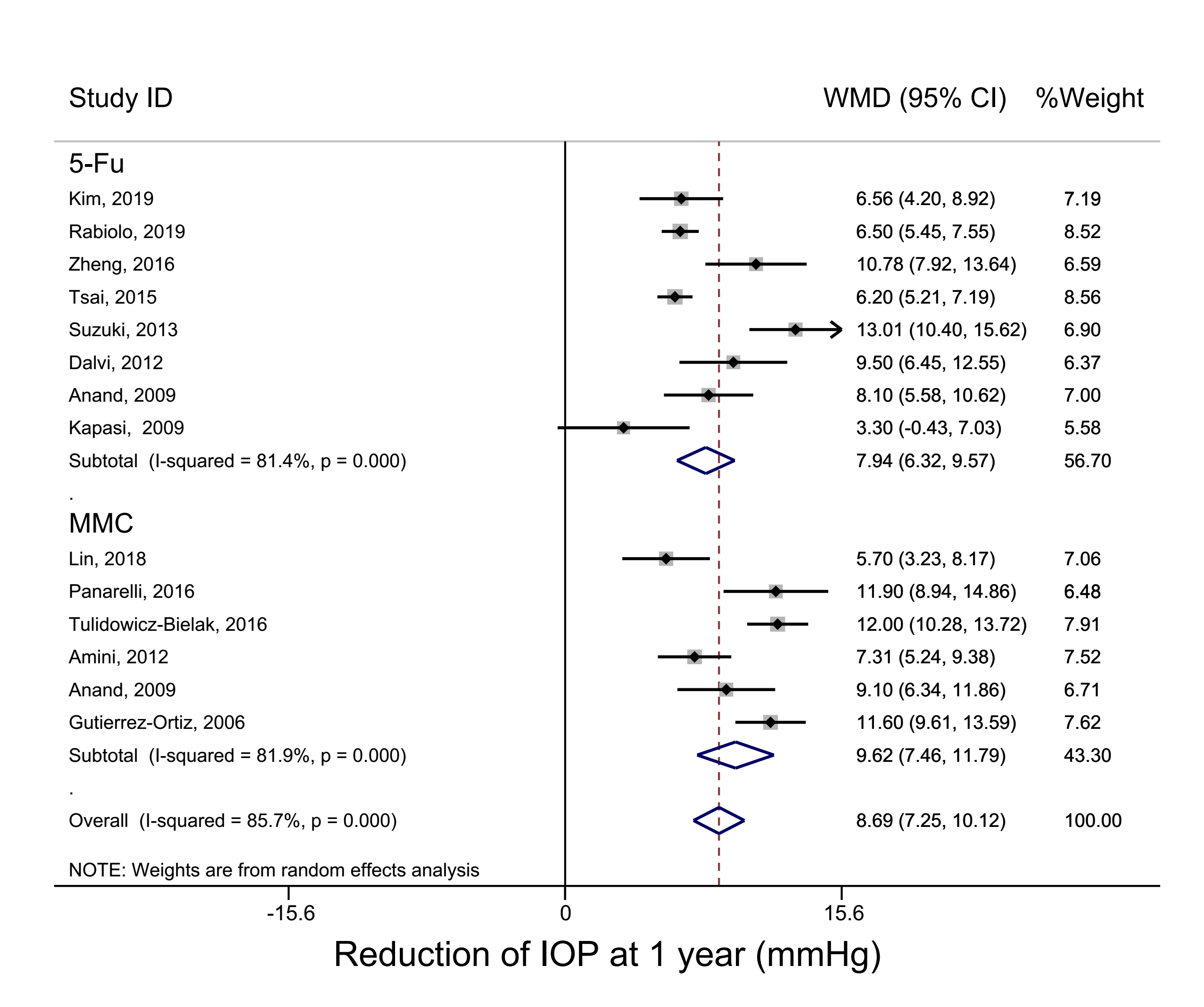


**Supplementary Figure 8** **Forest plots for WMD of IOP reduction at 1 year**: Weighted mean difference (WMD) of reduction in intraocular pressure (IOP) from baseline to 1 year. Subgroup analysis displayed the MMC and 5-Fu used as an adjuvant in bleb needling surgery.

# Supplementary Figure 9: Forest plot for WMD of IOP reduction at 2 years


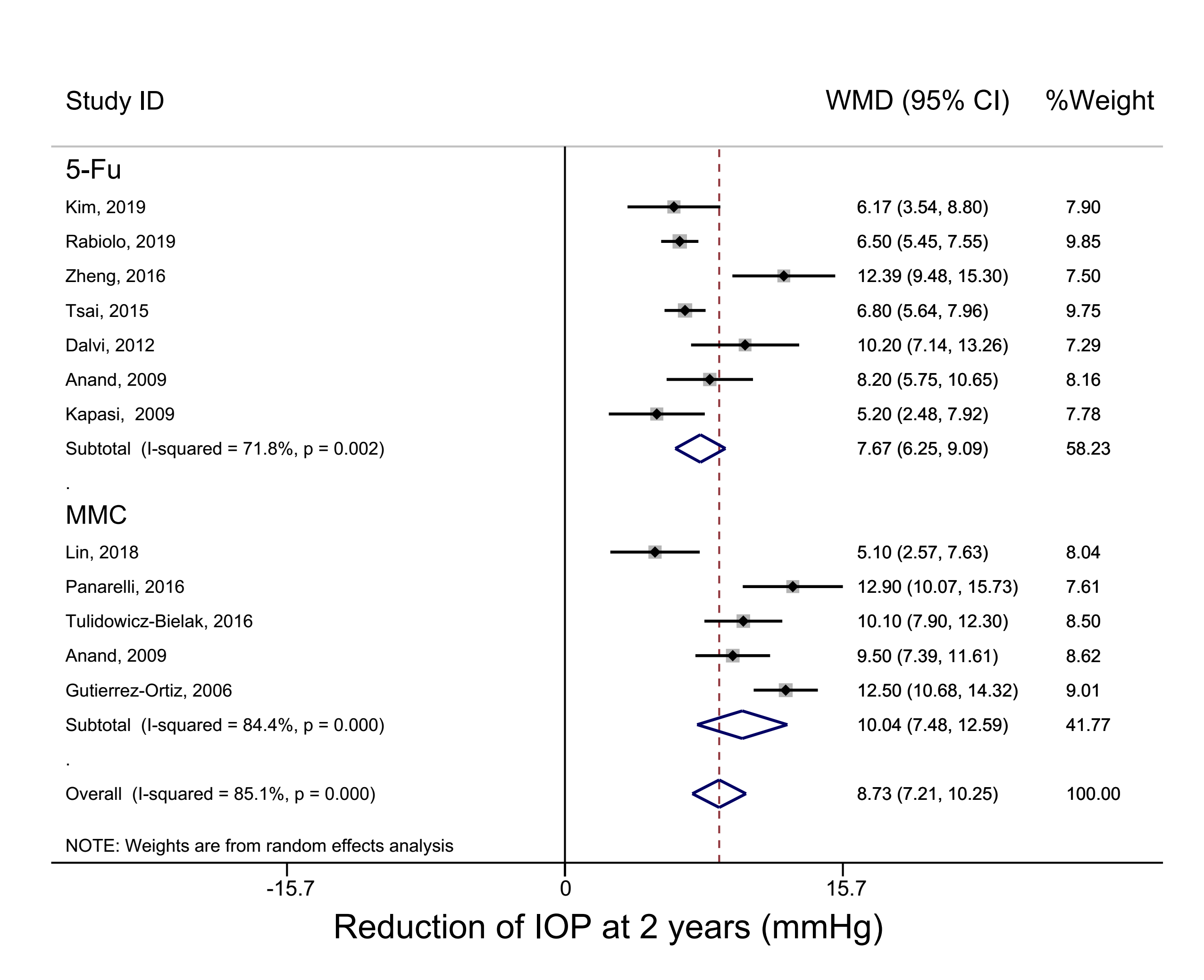


**Supplementary Figure 9 Forest plots for WMD of IOP reduction at 2 years**: Weighted mean difference (WMD) of reduction in intraocular pressure (IOP) from baseline to 2 years. Subgroup analysis displayed the MMC and 5-Fu used as an adjuvant in bleb needling surgery.

# Supplementary Figure 10: Forest plot for WMD of antiglaucomatous medication reduction at the last visit


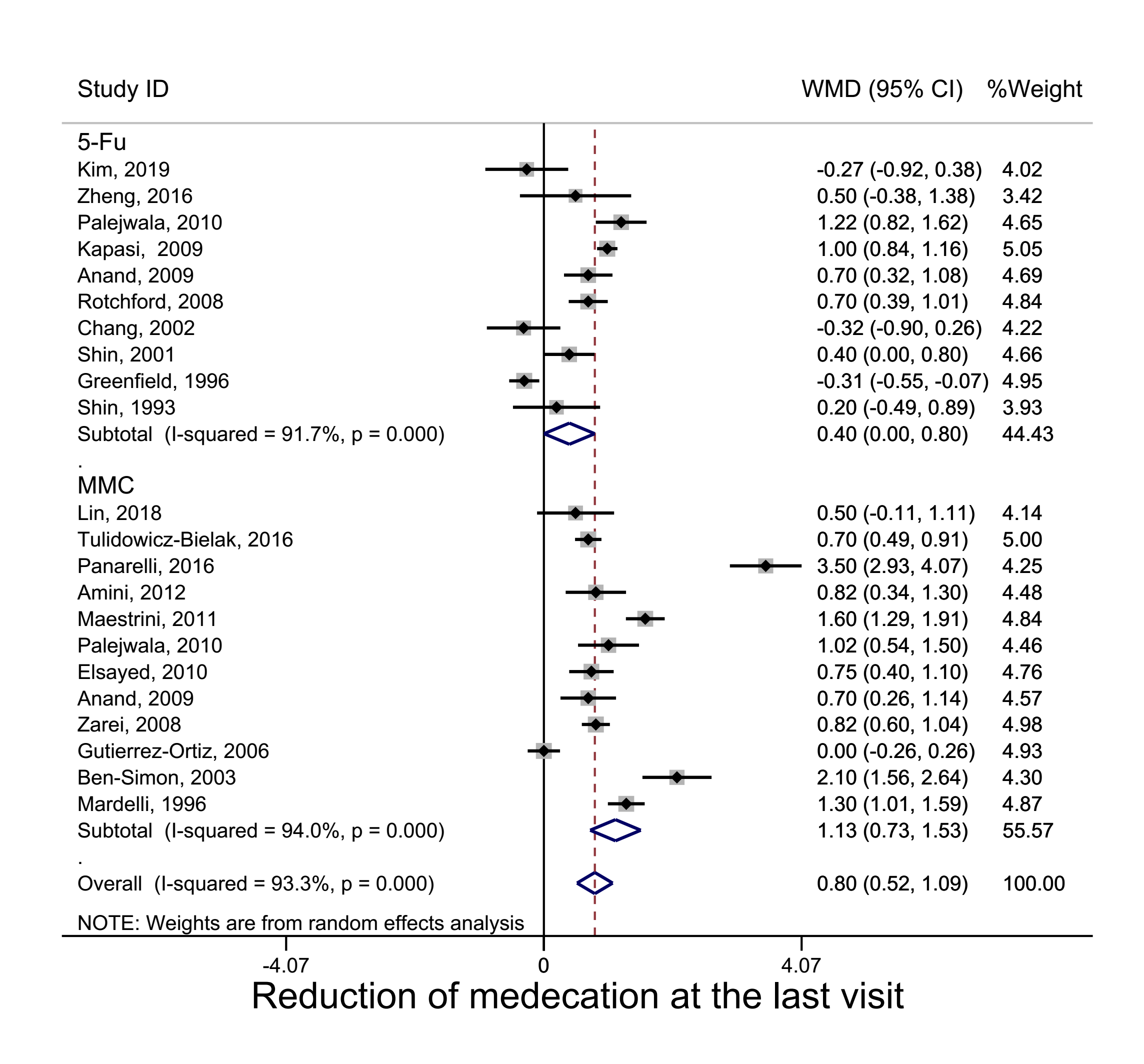


**Supplementary Figure 10** **Forest plots for WMD of antiglaucomatous medication reduction at the last visit**: Weighted mean difference (WMD) of reduction in topical antiglaucomatous medication from baseline to the last visit. Subgroup analysis displayed the MMC and 5-Fu used as an adjuvant in bleb needling surgery.

# Supplementary Figure 11: Sensitivity analysis for WMD of IOP reduction at last visit


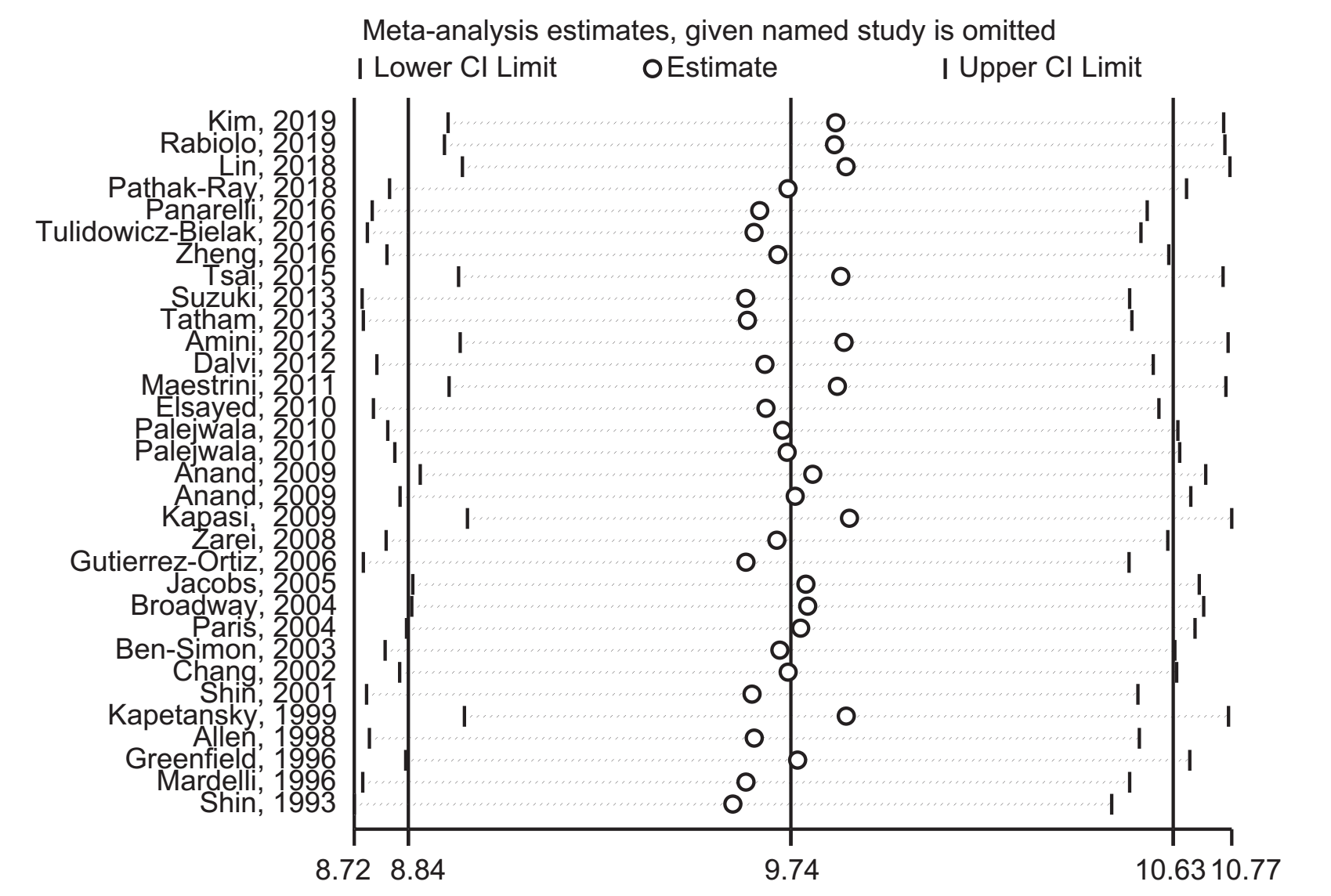


**Supplementary Figure 11** **Sensitivity analysis for WMD of IOP reduction at last visit:** The effect of a single trial on the overall pooled estimate was evaluated by omitting one trial in each turn. The estimate mainly fell in the range of 95% confidence interval, indicating that the model was stable.

# Supplementary Table 1: Detailed technique of bleb needling for each study

**Supplementary Table 1** Detailed technique of bleb needling for each study

| **Author, year** | **Antimetabolites with initial filtering procedure** | **Anaesthesia** | **Site of puncture** | **Distance from the flap (mm)** | **Entry underneath scleral flap** | **Injection of antimetabolites mixed with anaesthetic** | **Antimetabolites in needling** | **Dose** | **Concentration** | **Post needling use of steroids** |
| --- | --- | --- | --- | --- | --- | --- | --- | --- | --- | --- |
| **Rabiolo, 2019** | MMC: 157 (100.0) | Topical | Superotemporal/Superonasal | 5-10 | Yes | No | 5-Fu | 5mg | N/A | Subconjunctival/No |
| **Okka, 2019** | 5-Fu: 6 (37.5); None: 10 (62.5) | Topical | N/A | N/A | Yes | No | 5-Fu | 5mg | 25mg/ml | No |
| **Kim, 2019** | MMC: 25 (71.4); None: 10 (28.6) | Topical | N/A | 10 | Yes | No | 5-Fu | 5mg | 50mg/ml | Topical |
| **Than, 2018** | MMC: 70 (72.9); 5-Fu: 2 (2.1); None: 4 (4.2); Unknown: 12 (12.5) | Topical | Temporal | N/A | No | No | 5-Fu | 10mg | N/A | Topical |
| **Pathak-Ray, 2018** | N/A | Topical and Subconjunctival | Superior | 8-10 | Yes | Yes | MMC | 0.06mg | 0.3mg/ml | Topical |
| **Lin, 2018** | MMC: 24 (54.5); 5-Fu: 3 (6.8); None: 10 (22.7); Unknown: 2 (4.5) | Topical or Subconjunctival | Superior | N/A | Yes | No | MMC | 0.15–0.2 ml | 0.02% | Topical |
| **Zheng, 2016** | MMC: 27 (81.8); 5-Fu: 2 (6.1); Unknown: 4 (12.1) | Peribulbar or Subconjunctival | Superior | N/A | Yes | No | 5-Fu | N/A | N/A | Subconjunctival |
| **Tulidowicz-Bielak, 2016** | MMC: 121 (100.0) | Topical and Subconjunctival | Superior | 5-10 | Yes | No | MMC | 0.03mg | 0.3mg/ml | Topical |
| **Panarelli, 2016** | MMC: 19 (70.4); 5-Fu: 6 (22.2); Unknown: 2 (7.4) | Retrobulbar | Superior | 10 | Yes | No | MMC | 0.0167mg | 0.167mg/ml | Subconjunctival and Topical |
| **Liu, 2016** | MMC group: MMC 34 (85.0); None: 6 (15.0); 5-Fu group: MMC 29 (82.9); None: 6 (17.1) | Topical | N/A | >10 | Yes | No | MMC or 5-Fu | MMC: 0.02mg; 5-Fu: 5mg | MMC: 0.2mg/ml; 5-Fu: 50mg/ml | No |
| **Lee, 2016** | Unspecified antimetabolites: 35 (85.4); None: 2 (4.9); Unknown: 4 (9.8) | Topical | N/A | 2-3 from the bleb | Yes | No | 5-Fu | 5mg | 50mg/ml | No |
| **Tsai, 2015** | MMC: 227 (100.0) | Topical | N/A | N/A | Yes | No | 5-Fu | 5mg | 50mg/ml | Topical |
| **Tatham, 2013** | MMC: 18 (52.9); 5-Fu: 11 (32.4); None: 5 (14.7) | Topical or Peribulbar | N/A | 10 | Type-1 needling group: no; Type-2 needling group: Yes | No | 5-Fu | 7.5mg | 50mg/ml | Topical |
| **Suzuki, 2013** | MMC: 20 (100.0) | Topical | N/A | N/A | No | No | 5-Fu | 5mg | 25mg/ml | Topical |
| **Dalvi, 2012** | MMC: 20 (100.0) | Topical | N/A | N/A | Yes | No | 5-Fu | 5&10mg | 50mg/ml | Topical |
| **Amini, 2012** | MMC: 27 (100.0) | Topical | Temporal | 10 | Yes | No | MMC | 0.02mg | 0.2mg/ml | Topical |
| **Maestrini, 2011** | MMC: 31 (24.8); 5-Fu: 2 (1.6); MMC+5-Fu: 9 (7.2); None: 49 (39.2); Unknown: 34 (27.2) | Topical and Subconjunctival | Superior | As far as possible | No | Yes | MMC | 8μg | 0.08mg/ml | Topical |
| **Palejwala, 2010** | N/A | Topical and Subconjunctival | Temporal/ Nasal | 8 | Yes | No | MMC or 5-Fu | MMC: 0.04mg; 5-Fu；5mg | MMC: 0.2mg/ml; 5-Fu：50mg/ml | Anterior chamber injection and Topical |
| **Elsayed, 2010** | N/A | General and Subconjunctival | N/A | 5-8 | Yes | Yes | MMC | 0.004g | 0.04mg/ml | Topical |
| **Kapasi, 2009** | N/A | Topical | N/A | N/A | Yes | No | 5-Fu | 5mg | 50mg/ml | Topical |
| **Anand, 2009** | MMC group: MMC 23 (51.2); None: 22 (48.8); 5-Fu group: MMC 14 (26.4); 5-Fu: 3 (5.7); None: 36 (67.9) | Topical | Superior | 10 | No | No | MMC or 5-Fu | MMC: 0.02mg; 5-Fu: 5mg | MMC: 0.2mg/ml; 5-Fu：50mg/ml | Topical |
| **Zarei, 2008** | N/A | Topical | N/A | 6 | Yes | No | MMC | 0.1ml | 0.04% | Topical |
| **Rotchford, 2008** | MMC/5-Fu: 37 (45.7); None: 44 (54.3) | Topical | Temporal | 10 | Yes | No | 5-Fu | 5mg | N/A | Topical |
| **Gutierrez-Ortiz, 2006** | None: 34 (100.0) | Topical | N/A | 10 | Yes | No | MMC | 0.01ml | 0.02% | Topical |
| **Shetty, 2005** | MMC: 28 (63.6); 5-Fu: 2 (4.5); None: 14 (31.8) | Topical and Subconjunctival | Superior | N/A | Yes | Yes | MMC | 0.04mg | 0.2mg/ml | Topical |
| **Jacobs, 2005** | MMC: 9 (32.1); 5-Fu: 5 (17.9); None: 14 (50.0) | Topical | Superolateral | 10 | Yes | No | 5-Fu | 5mg | 50mg/ml | Topical |
| **Paris, 2004** | MMC: 28 (82.4); None: 6 (17.6) ^#^ | Retrobulbar and Lid | N/A | 8-10 | Yes | No | 5-Fu | 10mg | 50mg/ml | No |
| **Broadway, 2004** | MMC: 8 (7.9); 5-Fu: 45 (44.6); β-irradiation: 4 (4.0); None: 44 (43.6) | Topical | Temporal | 5-6 | Yes | No | 5-Fu | 5mg | 25mg/ml | Topical |
| **Ben-Simon, 2003** | N/A | Topical | N/A | 7 | Yes | No | MMC | 0.02mg/0.04mg | 0.2mg/ml; 0.4mg/ml | Topical |
| **Hawkins, 2002** | MMC: 36 (83.7); 5-Fu: 3 (7.0); None: 4 (9.3) | Topical and Subconjunctival | N/A | 8-10 | No | No | 5-Fu | 5mg | 50mg/ml | Topical |
| **Chang, 2002** | MMC: 11 (44.0); None: 14 (56.0) | Topical and Subconjunctival | N/A | N/A | No | No | 5-Fu | 5mg | N/A | No |
| **Shin, 2001** | N/A | Topical | N/A | 10 | Yes | No | 5-Fu | 5mg | 50mg/ml | Topical |
| **Kapetansky, 1999** | N/A | Topical and Subconjunctival | Temporal | N/A | No | No | MMC | 0.03mg | 0.3mg/ml | Topical |
| **Allen, 1998** | 5-Fu: 21 (65.6); None: 11 (34.4) | Topical | Temporal | 10 | No | No | 5-Fu | 5mg | 25mg/ml | Topical |
| **Mardelli, 1996** | MMC: 25 (40.3); 5-Fu: 16 (25.8); None: 21 (33.9) | Topical and Subconjunctival | Temporal/ Superior | 10 | Yes | Yes | MMC | 0.004mg | 0.4mg/ml | Topical |
| **Greenfield, 1996** | N/A | Topical | N/A | 5 | Yes | No | 5-Fu | 5mg | N/A | Topical |
| **Shin, 1993** | N/A | Topical | N/A | 10 | Yes | No | 5-Fu | 5mg | 10mg/ml | Topical |

* MMC: mitomycin C; 5-Fu: 5-fluorouracil. N/A meant that the data were not presented in the original article.

^#^ Number of patients was displayed.

# Supplementary Table 2: Subgroup analyses for WMD for IOP reduction at the last visit

**Supplementary Table 2** Subgroup analyses for WMD for IOP reduction at the last visit.

|  | **No. of study ID*** | **WMD** | **95% Lower limit** | **95% Upper limit** | ***I^2^* (%)** | |
| --- | --- | --- | --- | --- | --- | --- |
| **Overall** | 32 | 9.74 | 8.85 | 10.63 | 80.9 | |
| **Subgroup analyses** | | | | | |  |
| **Medication** | | | | | |  |
| **5-Fu** | 18 | 9.75 | 8.56 | 10.94 | 79.9 | |
| **MMC** | 14 | 9.72 | 8.41 | 11.03 | 81.6 | |
| **Bleb morphology** | | | | | |  |
| **Flat** | 1 | 6.90 | 5.40 | 8.40 | N/A | |
| **Encapsulated** | 3 | 12.29 | 10.56 | 14.02 | 0 | |
| **Not defined** | 27 | 9.61 | 8.67 | 10.55 | 80.6 | |
| **Acceptance for repeating needling** | | | | | |  |
| **Yes** | 23 | 9.45 | 8.37 | 10.52 | 82.2 | |
| **No** | 9 | 10.47 | 9.07 | 11.86 | 68.7 | |
| **Surgery condition** | | | | | |  |
| **Slit lamp** | 11 | 8.98 | 7.43 | 10.53 | 81.1 | |
| **OR** | 17 | 10.10 | 8.96 | 11.24 | 76.4 | |
| **Not defined** | 4 | 10.53 | 7.17 | 13.88 | 88.3 | |
| **Region** | | | | | |  |
| **Asia-Pacific** | 9 | 8.78 | 7.21 | 10.35 | 75.1 | |
| **Europe** | 10 | 9.77 | 8.15 | 11.39 | 83.6 | |
| **America** | 13 | 10.18 | 8.56 | 11.80 | 81.2 | |
| **Africa** | 1 | 11.27 | 9.75 | 12.79 | N/A | |
| **Study design** |  |  |  |  |  | |
| **RCT** | 1 | 13.01 | 10.40 | 15.62 | - | |
| **Prospective** | 4 | 10.00 | 7.41 | 12.58 | 87.1 | |
| **Retrospective** | 27 | 9.58 | 8.60 | 10.55 | 80.1 | |

*32 groups from 30 studies were counted in number of study ID.

^#^ IOP: intraocular pressure; MMC: mitomycin C; OR: operating room; RCT: randomized control study; WMD: weighted mean difference; 5-Fu: 5-fluorouracil

# Supplementary Table 3: Meta-regression for WMD for IOP reduction at the last visit

**Supplementary** **Table 3** Meta-regression with different covariates explained part of the heterogeneity sources of WMD of IOP reduction at the last visit.

| **Covariates** | ***I*^2^_res_ (%)** | **Adj R^2^(%)** | **p** |
| --- | --- | --- | --- |
| IOP before bleb needling | 56.8 | 64.9 | <0.001 |
| Sample size | 75.1 | 10.7 | 0.099 |
| Publication year | 77.8 | 10.1 | 0.088 |
| Age | 81.4 | -3.7 | 0.608 |
| No. of high-risk domain in AHRQ | 80.6 | -3.4 | 0.726 |
| Interval between trabeculectomy and bleb needling | 82.4 | -4.3 | 0.526 |

**I*^2^_res_: residual heterogeneity after meta-regression; Adj R^2^: proportion of between-study variance explained by meta-regression
